# Accumulation of alpha-synuclein within the liver, potential role in the clearance of brain pathology associated with Parkinson’s disease

**DOI:** 10.1186/s40478-021-01136-3

**Published:** 2021-03-20

**Authors:** Juan F. Reyes, Sara Ekmark-Léwen, Marina Perdiki, Therése Klingstedt, Alana Hoffmann, Emilia Wiechec, Per Nilsson, K. Peter R. Nilsson, Irina Alafuzoff, Martin Ingelsson, Martin Hallbeck

**Affiliations:** 1 Department of Biomedical and Clinical Sciences (BKV), Linköping University, 581 85 Linköping, Sweden; 2 Department of Clinical Pathology, Linköping University, 581 85 Linköping, Sweden; 3 Department of Public Health and Caring Sciences, Section of Geriatrics, Uppsala University, Uppsala, Sweden; 4 Department of Physics, Chemistry and Biology, Linköping University, 581 83 Linköping, Sweden; 5 Department of Molecular Neurology, University Hospital Erlangen, Friedrich-Alexander-Universität Erlangen-Nürnberg, Erlangen, Germany; 6 Department of Otorhinolaryngology, Anesthetics, Operations and Special Surgery Center, Linköping University, 581 85 Linköping, Sweden; 7 Department of Neurobiology, Care Sciences and Society, Center for Alzheimer Research, Division of Neurogeriatrics, Karolinska Institutet, 171 64 Solna, Sweden; 8 Department of Pathology, Uppsala University Hospital, Uppsala, Sweden

## Abstract

Alpha-synuclein (α-syn) aggregation is the hallmark pathological lesion in brains of patients with Parkinson’s disease (PD) and related neurological disorders characterized as synucleinopathies. Accumulating evidence now indicates that α-syn deposition is also present within the gut and other peripheral organs outside the central nervous system (CNS). In the current study, we demonstrate for the first time that α-syn pathology also accumulates within the liver, the main organ responsible for substance clearance and detoxification. We further demonstrate that cultured human hepatocytes readily internalize oligomeric α-syn assemblies mediated, at least in part, by the gap junction protein connexin-32 (Cx32). Moreover, we identified a time-dependent accumulation of α-syn within the liver of three different transgenic (tg) mouse models expressing human α-syn under CNS-specific promoters, despite the lack of α-syn mRNA expression within the liver. Such a brain-to-liver transmission route could be further corroborated by detection of α-syn pathology within the liver of wild type mice one month after a single striatal α-syn injection. In contrast to the synucleinopathy models, aged mice modeling AD rarely show any amyloid-beta (Aß) deposition within the liver. In human post-mortem liver tissue, we identified cases with neuropathologically confirmed α-syn pathology containing α-syn within hepatocellular structures to a higher degree (75%) than control subjects without α-syn accumulation in the brain (57%). Our results reveal that α-syn accumulates within the liver and may be derived from the brain or other peripheral sources. Collectively, our findings indicate that the liver may play a role in the clearance and detoxification of pathological proteins in PD and related synucleinopathies.

**Supplementary Information:**

The online version contains supplementary material available at 10.1186/s40478-021-01136-3.

## Introduction

The progressive accumulation of protein deposits comprised primarily of the alpha-synuclein (α-syn) protein are key signature lesions of Parkinson’s disease (PD), dementia with Lewy bodies (DLB) and multiple system atrophy (MSA), related neuropathological disorders collectively known as synucleinopathies [1]. While the clinical manifestations of MSA and DLB are highly variable among patients [2], PD is typically characterized by a progressive decline of motor and non-motor functions which correlate with disease progression [3, 4]. Pathologically, PD and DLB are characterized primarily by the accumulation of α-syn within neurons while in MSA, α-syn accumulation is observed mainly in cells of the oligodendrocyte lineage [5]. In PD, rare genetic mutations (A30P, E46K, H50Q, G51D, A53E/T) or multiplications of the α-syn gene (*SNCA*) have been genetically linked to autosomal dominant forms of PD [6–8]. Although the accumulation of α-syn in the brain is the pathological hallmark of PD, recent evidence suggests that α-syn accumulation is not limited to the brain as it also appears within multiple organs outside the central nervous system (CNS). Indeed, several studies have now identified α-syn accumulation within the retina, skin, heart, gastrointestinal region, appendix and colon (for review see [9, 10]). Interestingly, α-syn has also been found in body fluids including blood, cerebrospinal fluid, saliva, and most recently in tear fluid [11–16]. These findings provide further evidence for PD as a multisystem disorder and strengthen the hypothesis that spreading of α-syn pathology between peripheral organs and the brain may be an early and critical feature in PD pathogenesis.

Accumulating evidence suggesting that α-syn pathology can spread from the brain to the periphery or from the gut to the brain has provided support for the ‘dual hit’ hypothesis in PD [17]. Multiple studies have demonstrated that injections of α-syn into the olfactory bulb leads to a retrograde spreading of α-syn pathology from the site of injection to multiple brain regions over time [18–20]. Midbrain overexpression of human α-syn was also demonstrated to reach the gastric wall where it can accumulate into preganglionic vagal terminals [21]. The existence of a gut to brain route is strongly supported by early gastrointestinal symptoms which appear many years prior to neurodegeneration [22, 23]. A recent study also suggests that removal of the appendix can reduce PD risk and delay the age of symptom onset [24], although other studies have reported no such association [25]. In animal models, recent studies have shown that injection of α-syn within the gut leads to α-syn accumulation in the brain [26]. Additionally, mouse truncal vagotomy following α-syn injection in the gut was recently shown to prevent α-syn spread to the brain and, therefore, the neurodegenerative process associated with PD [27]. Notably, a recent study has suggested the existence of two PD subtypes, a brain-first or a body-first subtype, indicating that α-syn pathology may spread from brain to the enteric or peripheral nervous system (PNS) or vice versa [28]. However, a bidirectional spread of pathology has also been reported [29, 30].

In our recent work, we identified the gap junction protein connexin-32 (Cx32) centrally involved in the uptake and propagation of α-syn oligomers (oα-syn) in neurons and oligodendrocytes [31], the primary cell types affected by α-syn aggregation in PD and MSA, respectively [5, 32]. Curiously, aside from the brain, Cx32 is highly expressed within normal liver hepatocytes [33], the main cell type within the liver and the organ responsible for substance clearance and detoxification.

In the current study, we investigated whether the liver is susceptible to α-syn accumulation in PD. We demonstrate for the first time that primary human hepatocytes show a selective uptake for oligomeric vs. fibrillar α-syn assemblies in vitro. In situ, we found an age-dependent accumulation of human α-syn pathology within the liver in animal models of PD expressing either human wild type (L61), or mutant (A30P) α-syn [34, 35]. Interestingly, we could also detect the presence of human α-syn within the liver of mice expressing human α-syn within oligodendrocytes modeling MSA (MBP29) [36]. We also demonstrate that α-syn accumulation in the liver is not due to local hepatic mRNA expression thus indicating that in these synucleinopathy models, α-syn is derived from the brain. We further characterized these inclusions using an array of highly specific human α-syn antibodies including the phospho-specific antibody targeting α-syn at serine 129 (pS129). The aggregation state of these inclusions was further validated using Thioflavin T and luminescent conjugated oligothiophenes (LCOs), conformational sensitive molecular ligands validated to bind to a wider range of protein aggregates compared to conventional ligands [37–41]. In human liver tissue, we identified cases with neuropathologically confirmed α-syn pathology containing α-syn within the hepatocellular structures to a higher degree (75%) than control subjects without α-syn accumulation in the brain (57%). Taken together, our results suggest a liver involvement in the clearance of pathological α-syn assemblies.

## Materials and methods

### Generation of ATTO-labeled α-syn

Recombinant α-syn was purchased from Alexotech in a lyophilized form. To covalently label α-syn with ATTO-550/488 fluorescent tag, we mixed proteins with the reactive ATTO dye freshly dissolved in DMSO (Sigma-Aldrich) as previously reported [42]. Unreactive dye was removed by gel filtration chromatography using GE Sephadex G-25 (GE Healthcare), and labeled fractions were analyzed by Western blot analysis and size exclusion chromatography (SEC) as previously reported [31, 42]. Positive fractions were pooled, lyophilized and then stored at − 20 °C until further use.

### Generation of human and mouse α-syn assemblies

To generate α-syn assemblies, we solubilized ATTO-labeled proteins in PBS to generate soluble monomers at a concentration of 4.0 mg/mL. To generate α-syn oligomers or fibrils, we incubated α-syn monomers at 37 °C for 5 days in an Eppendorf SS mini-shaker (Eppendorf) with constant shaking at either 350 or 1000 RPM, respectively. Assembled proteins were then aliquoted and stored at -80 °C for further analysis.

### Transmission electron microscopy (TEM) characterization of α-syn assemblies

α-syn oligomers and fibrillar assemblies were prepared for TEM analysis as previously reported [31, 42]. Briefly, each sample was placed on carbon-coated 200-mesh grids, negatively stained with 1% uranyl acetate and analyzed with a Jeol JEM1230 (Jeol). Images were taken using a Gatan Orius CCD camera (Gatan).

### Primary human hepatocytes and generation of HuH-Cx32 hepatocyte cell line

Primary human hepatocytes were obtained from 5 different human patients of both sexes containing 6 × 10^6^ cells per vial. These "5-Donors" were purchased in a plateable form and plated according to manufacturer’s instructions (Thermo Scientific). To express Cx32 in HuH-7 cells [43], and generate a stable cell lines, we transfected cells with plasmid vectors expressing human Cx32-mCherry (a gift from Michael Davidson, Addgene plasmid # 55022) according to the manufacturer's instructions (Amaxa Nucleofector, Lonza). Transfected cells were then selected with appropriate antibiotics (G418, Sigma-Aldrich) followed by single cell sorting using FACS into 96-well plates to generate clonal cell lines.

### α-Syn hepatocyte treatment

Primary and HuH-7 hepatocytes stably expressing Cx32 were treated with 1 µM oligomeric or fibrillar α-syn assemblies in serum free MEM medium (with 1% glutamine and 1% penicillin/streptomycin) for 24 h.

### Semi-quantitative real-time PCR (qRT-PCR)

RNA was extracted from cell and tissue samples using AllPrep DNA/RNA Universal Kit according to the manufacturer’s instructions (Qiagen). cDNA was generated from RNA using a High- Capacity RNA to cDNA Kit (Applied Biosystems). Each RT-PCR reaction utilized 10 ng cDNA and was analyzed in technical duplicates. Reactions were analyzed on a 7500 Fast Real-Time PCR System (Applied Biosystems). All primer/probes used in this study utilized the FAM–MGB TaqMan system and were purchased from Applied Biosystems or were generated using the primer design tool Primer-BLAST (http://www.ncbi.nlm.nih.gov/ tools/primer-blast) (Additional file 10: Table I). Amplification of GAPDH was used as an internal standard. The Ct method was used to determine the fold-difference in expression levels relative to a control sample.

### Western blot analysis

Following α-syn treatment, cells were washed three times in PBS for 5 min then incubated with TrypLE Express™ (Gibco) for 3–5 min at room temperature. Cells were pelleted by centrifugation at 13,000 RPM (Eppendorf) and lysed in RIPA buffer followed by a brief sonication to completely lyse cell pellets and determine protein concentration using DC Assay (Bio-Rad). All samples were resuspended in a final concentration of 1X Laemmli’s sample buffer, boiled for 5 min, separated on 4–20% SDS-PAGE (CBS Scientific) and transferred to nitrocellulose membranes using iBLOT kits (Life Technologies). Nonspecific protein binding was blocked by incubating membranes with 2% nonfat dry milk, followed by incubation with primary antibodies at 4 °C overnight (Additional file 10: Table I). After rinsing the membranes using Tween-tris-buffered saline (Medicago), we incubated them with peroxidase-conjugated goat anti-mouse or anti-rabbit IgG H + L secondary antibodies (Dako) for 1 h at RT, followed by ECL substrate (Bio-Rad) to visualize the signal on a ChemiDoc XRS + (Bio-Rad). Densitometric analysis was performed using ImageJ (Fiji), and values with arbitrary units were normalized to the signals obtained from total protein measured with loading controls (ß-actin/GAPDH).

### Immunoprecipitation of α-syn

α-syn proteins were precipitated according to a previous report [44]. Briefly, cell lysates were homogenized in lysis buffer containing 20 mM Tris–HCl (pH 7.9), 137 mM NaCl, 5 mM N Na_2_EDTA, 1 mM EGTA, 10% glycerol, and 1% Triton X-100 with protease and phosphatase inhibitors (Roche). 500 µg of cell lysates were incubated with 1 µg of 14H antibody overnight at 4 °C. A 1:1 suspension of Protein A/G agarose beads was added (Thermo Scientific), and the mixture was incubated at 4 °C for 4 h. The beads were then pelleted and washed thoroughly with cell lysis buffer according to the manufacturer’s instructions. Bound proteins were separated by SDS-PAGE followed by Western blot analysis.

### Animals and tissue samples

Male mice used for this study were anesthetized with isoflurane before being transcardially perfused with 0.9% saline. After perfusion, the brain and liver were isolated then fixed in 4% PFA, followed by 70% ethanol before the tissue was paraffin embedded. Samples from young (3-month, control *n* = 7, tg *n* = 8) or aged (18-month, control: *n* = 7, tg: *n* = 12) male homozygous (Thy-1)-h[A30P] α‐syn tg and wild type (WT) control mice were isolated [34]. Similarly, brain and livers from young (3-month, control: *n* = 4, tg *n* = 6) and aged male mice (12-month, WT control: *n* = 4, tg: *n* = 8) expressing the human wild type form of α‐syn (Thy-1-L61) were also used for this study. For tg mice modeling MSA (MBP29) [45], only brain and livers from 4-month-old mice were used (control: *n* = 4, tg: *n* = 4). For *App* knock-in mice harboring Beyreuther/Iberian mutation (*App*^*NL−F*^ mice) modeling AD, only brain and aged livers were used (20 and 24 months, WT *n* = 4, *App*-knock-in: *n* = 4 and 2, respectively). All animals were housed in open cages on a 12:12 h reversed dark: light cycle in a temperature‐ and humidity‐controlled room and properly cared by the animal facility. All experiments involving mice were approved by the Local Animal Ethics Committees. The use and care of the animals were conducted in accordance with the EU Directive 2010/63/EU for animal experiments.

### Striatal injection of α-syn in wild type mice

The oα-syn preparations were diluted into sterile PBS and sonicated briefly before intracerebral injection. Male C57bl/6 N mice (3 months of age, *n* = 4) were used for the study. Mice were anesthetized with isoflurane and were stereotactically injected with 5 μg total protein per brain (total injection volume 2 μl). Material was injected with a Hamilton syringe at a rate of 0.1 μl per min into the right dorsal striatum (coordinates: + 0.2 mm relative to bregma, 2.0 mm from midline) at a depth of 2.6 mm below the dura, with the needle in place for 10 min at target. After recovery from surgery, animals were monitored regularly for weight loss and general health status. Animals were sacrificed at 1-month post-protein injection by isoflurane anesthesia followed by transcardial perfusion with 0.9% saline. Brains and livers were then isolated and fixed in 4% PFA, followed by 70% ethanol and subsequent paraffin embedding as described above.

### Immunocytochemistry and immunohistochemistry

Primary and HuH-7 hepatocytes were processed for immunocytochemistry or immunohistochemistry as previously reported [42]. Briefly, cells fixed with 4% PFA were washed three times with PBS and permeabilized by incubation in 0.1% Triton X-100 in PBS for 20 min on ice. Next, cells were washed with PBS and incubated in 5% BSA for 1 h at RT, followed by incubation with primary antibodies overnight at 4 °C (Additional file 10: Table I). The next day, we washed the cells with PBS and incubated them with respective fluorescently labeled secondary antibodies for 1 h (goat anti-mouse or goat anti-rabbit IgG Alexa Fluor 488, 564 or 633, Life Technologies), followed by Hoechst/DAPI staining (Sigma-Aldrich). For immunohistochemistry, paraffin-embedded liver tissue sections from mouse or human were rehydrated in xylene (Sigma-Aldrich) followed by incubation in decreasing concentrations of ethanol (100, 95, 70 and 50%) then rinsed in water for 10 min. Epitope retrieval was performed using acidic conditions according to the manufacturer’s instructions (Dako). Tissue sections were rinsed three times for 5 min in PBS, then incubated with 0.04% Triton X-100 in PBS for 1 h and processed for immunohistochemistry as described above followed by Sudan Black staining to eliminate lipofuscin autofluorescence as reported previously [46–48].

### LCO staining

p-FTAA and HS-68 were synthesized as described previously [38, 49] and tissue staining was performed as previously reported with slight modifications [37]. Briefly, paraffin-embedded liver tissue sections from mouse were rehydrated in xylene (Sigma-Aldrich) followed by incubation in decreasing concentrations of ethanol (100, 95, 70 and 50%) and rinsed in water for 10 min. Tissue sections were rinsed three times for 10 min in PBS, then incubated with 0.04% Triton X-100 in PBS for 1 h and then stained with p-FTAA or HS-68 LCOs diluted to 3 µM in PBS for 30 min at RT. The spectral analysis of the HS-68 LCO was performed using an inverted Zeiss LSM 780 confocal microscope (Zeiss) exciting the ligand at 458 nm.

### Confocal image analyses

To visualize the fluorescence immunostaining, we used a Zeiss LSM 700 confocal microscope equipped with diode lasers (405-,488-,555-, or 639-lasers) used for excitation to acquire Z-stacks of consecutive confocal images. Overview images were taken using a Zeiss LSM 780 equipped with 405-, 488-, 559-, and 633 nm laser lines or a LSM 700 (see above) confocal laser-scanning microscope or with a Leica SP5 TCS MP inverted point-scanning confocal equipped with multiphoton for two photon excitations (710–1040 nm) equipped with a digital camera for widefield imaging.

### Statistical analyses

Statistical analyses were performed using a two-tailed unpaired Student’s *t*-test when comparing two genotypes or two-way ANOVA with Tukey’s test when comparing multiple samples and genotypes. For Western blot analyses, band intensities were quantified using ImageJ software (Fiji) software, and values with arbitrary units were normalized to the signal obtained from the protein loading control. In each data group, the results are expressed as the mean ± SEM. For semi-quantitative RT-PCR, statistics were calculated using 2^−ddCt^ values, using One-way ANOVA with Tukey analysis for multiple comparisons. All data analyses were performed with GraphPad Prism 7.0 (La Jolla, CA). Findings were regarded as significant when *p < 0.05, ****p < 0.0001.

## Results

### Primary human hepatocytes take up oligomeric α-syn assemblies in vitro

**Fig. 1:**
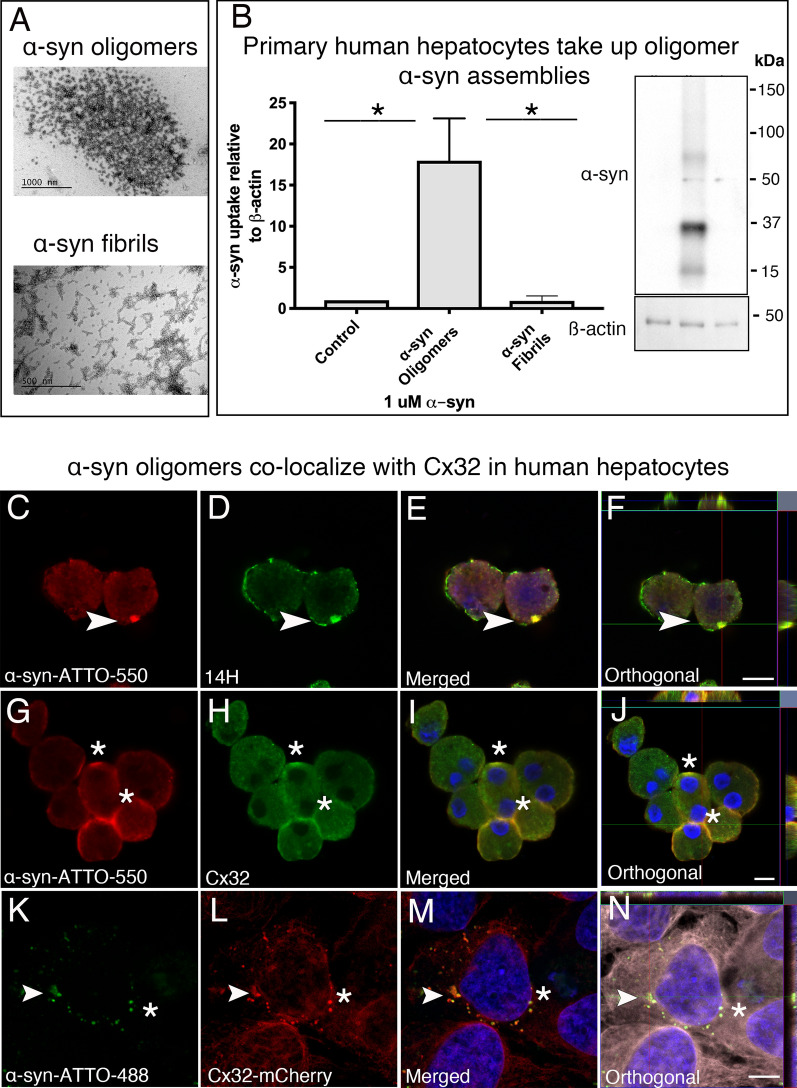
Human hepatocytes take up oligomeric α-syn assemblies in vitro. **A** Characterization of oligomeric and fibrillar α-syn-ATTO-550 assemblies using electron microscopy. **B **Western blot quantification of oligomeric and fibrillar α-syn uptake in primary human hepatocytes. **C**–**F** Immunocytochemistry of primary hepatocytes showing the internalization of α-syn-ATTO-550 labeled oligomers immunolabeled with the human specific α-syn antibody 14H (arrow head). **G, H** Immunocytochemistry of primary hepatocytes showing a partial co-localization between α-syn-ATTO-550 oligomers and the gap junction protein Cx32 (asterisk). **K–N** HuH-7 cells labeled with ß-tubulin (gray) expressing Cx32-mCherry (red) co-localize with α-syn-ATTO-488 oligomers (green, arrow head). All cells were counterstained with DAPI (blue). Bars = 20 µm. Statistical analysis was performed using one-way ANOVA with n = 3 using a Tukey's multiple comparison test, *p < 0.05

Given the abundant expression of Cx32 within liver hepatocytes and its recently identified role in promoting oligomeric α-syn uptake, we assessed whether the liver is susceptible to α-syn accumulation in PD. Therefore, we first investigated whether human hepatocytes could take up α-syn protein assemblies associated with PD [42]. Thus, we generated recombinant human α-syn oligomers (oα-syn) and fibrillar assemblies tagged to ATTO-550 and then characterized their ultrastructure using transmission electron microscopy [42]. Consistent with previous studies [31, 42], we identified the characteristic donut-like and filament ultrastructure typical for oα-syn and fibrillar assemblies, respectively, confirming that ATTO-550 has no discernible effect on assembly formation as previously reported (Fig. 1A) [31, 42]. Following protein assembly validation, we incubated oα-syn and non-sonicated fibrillar assemblies (to avoid oligomer formation [50]) with primary human hepatocytes isolated from five healthy adult human livers for 24 h. Subsequently, we assessed protein uptake by Western blot analysis and confocal imaging techniques. Consistent with previous results obtained from human neurons and oligodendrocytes [31], primary human hepatocytes in vitro displayed a preferential uptake for oα-syn assemblies compared to its fibrillar counterpart (Fig. 1B).

To further assess oα-syn uptake in primary human hepatocytes, we immunolabeled oα-syn with a pan-specific human α-syn antibody (14H2L1 (14H)) targeting the C-terminal region of the α-syn molecule (amino acids 121–140) (Additional file 10: Table I). As expected, we observed hepatocytic α-syn uptake localized intracellularly and at the cellular membrane as visualized by the ATTO-550 tag likely within a Cx32 gap junction plaque (Fig. 1C–F, arrow). Moreover, a direct co-localization between ATTO-550 and the human specific α-syn antibody 14H was clearly observed (Fig. 1C–F). We next immunolabeled the cells with Cx32 antibody and observed a partial co-localization between ATTO-550 labeled oα-syn assemblies and Cx32. This feature was particularly evident at the cellular membrane, the predominant subcellular localization of Cx32 (Fig. 1G–J, Additional file 1: Fig. 1A–L), thus validating a direct interaction between oα-syn and Cx32 during cellular uptake as previously reported [31]. Notably, in the absence of oα-syn treatment, no ATTO-550 fluorescence or α-syn immunoreactivity was observed, indicating cellular protein uptake and, importantly, validating the specificity of the antibody to human α-syn (Additional file 2: Fig. 2A–D). To further demonstrate a Cx32/α-syn protein interaction, we stably overexpressed Cx32-mCherry in the widely used human hepatocyte cell line HuH-7, a cell type that normally lacks Cx32 expression [51]. Following stable Cx32 protein expression, we next incubated HuH-7-Cx32 cells with ATTO-488-oα-syn protein assemblies for 24 h and then assessed protein uptake by confocal image analyses. Consistent with the results above, we observed protein internalization and most notably, a direct co-localization between Cx32 and ATTO-488 labeled oα-syn assemblies in human HuH-Cx32 cells (Fig. 1K–N). As expected, untreated cells showed no oα-syn reactivity (Additional file 2: Fig. 2E–H). We next immunoprecipitated (IP) α-syn from HuH-Cx32 cells treated with oα-syn for 24 h. Confirming the results above, IP of α-syn successfully pulled down Cx32 from HuH-Cx32 cells whereas untreated cells showed no Cx32 pull down (Additional file 2: Fig. 2I). It is worth noting that in contrast to unmodified cells, Cx32 expression in HuH-7 cells changed the cellular phenotype resembling a hepatocytic-like morphology, a signaling mechanism likely enhanced by the gap junction protein Cx32 (Additional file 2: Fig. 2J).

To determine whether human hepatocytes could degrade oα-syn following treatment, we incubated HuH-7 wild type or Cx32-expressing human hepatocytes with oα-syn assemblies for up to 72 h followed by assessment of protein uptake at different time points using Western blot analysis. Consistent with previous results [31], Cx32 expression in HuH-7 cells increased oα-syn uptake thus suggesting that Cx32 at the cellular membrane interacts with oα-syn and promotes intracellular uptake (Additional file 3: Fig. 3A–C) [31]. Notably, and consistent with the role of hepatocytes in toxin clearance and detoxification, wild type as well as Cx32 expressing HuH-7 hepatocytes showed a clear reduction of oα-syn assemblies over time (compare 24 h vs 72 h, Additional file 3: Fig. 2A, B). These results validate the degradation capacity of human hepatocytes to toxic substances including PD-associated pathology.

### Identification of human α-syn pathology within the liver of the A30P mouse model of PD

**Fig. 2:**
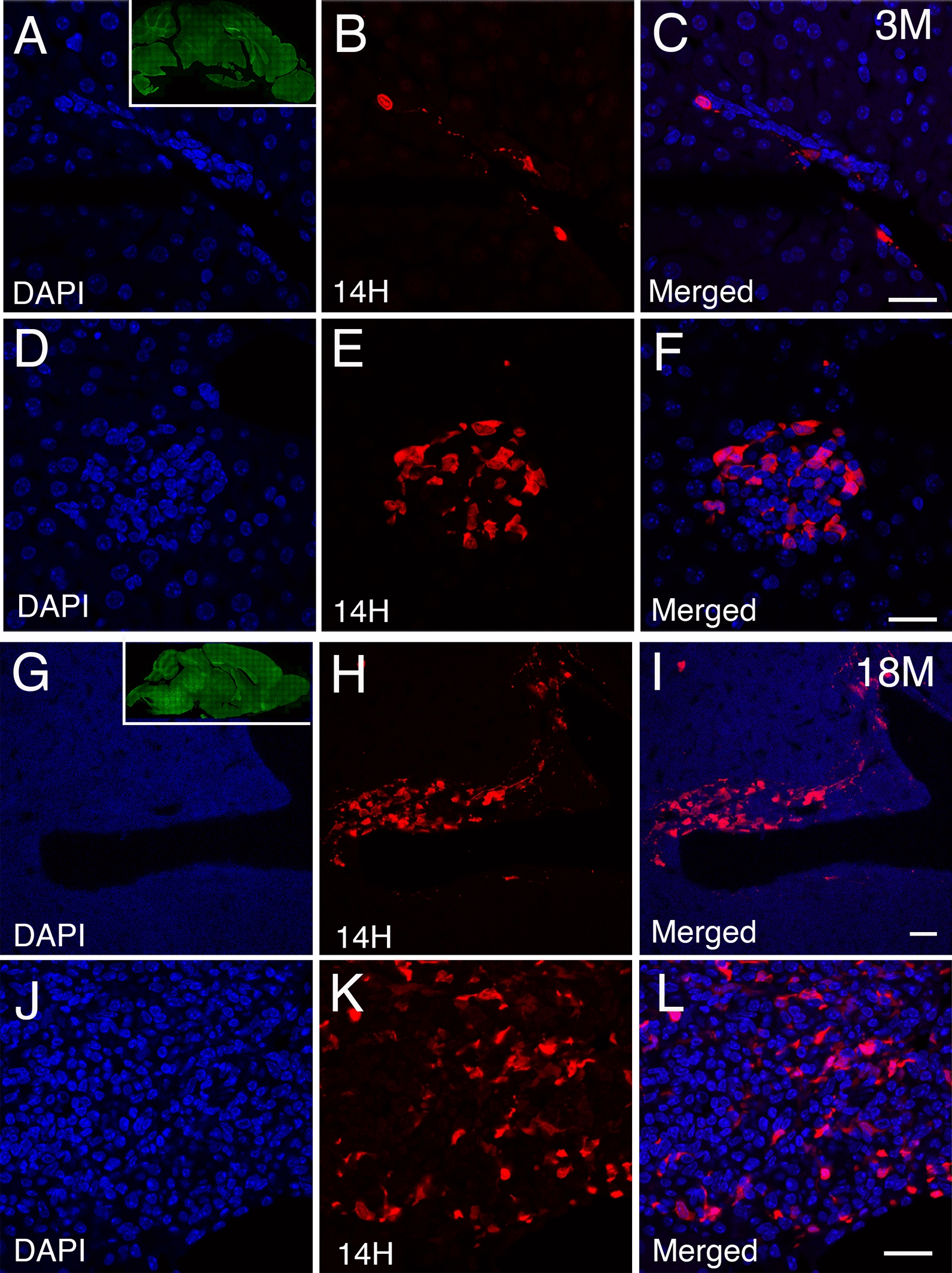
Age dependent accumulation of human α-syn deposits within the liver of the A30P transgenic mouse model. **A**–**C** Identification of human α-syn deposits (red) in young (3 months) liver tissue sections of the A30P mice located within the portal tracts and **D**–**F**, rosette-like structures within the liver parenchyma. Insert within panel A shows the deposition of α-syn at 3 months of age immune-stained with pS129 antibodies (green). **G**–**I** Aged A30P liver tissue sections (18 months) showing the progressive accumulation of α-syn deposits within the portal tracts and **J–L**, liver parenchyma. Insert within panel G shows the deposition of α-syn at 18 months of age immune-stained with pS129 antibodies (green). All liver tissue sections were counterstained with DAPI (blue). Bars = 20 µm

To assess whether the liver is susceptible to the accumulation of α-syn pathology in vivo, we turned to a widely used mouse model of PD overexpressing approximately 2 times the levels of human α-syn compared to mouse α-syn harboring the Ala30Pro mutation (A30P) under control of the neuronal Thy-1 promoter (Additional file 11: Table II) [52]. Thus, we performed immunohistochemistry on brain and liver tissue sections from young (3 months) and aged (18 months) transgenic (Tg) PD mice using human specific α-syn antibodies (Additional file 10: Table I). As expected, we observed a clear accumulation of human α-syn in the brain of this model between 3 and 18 months of age (Fig. 2A, G, inserts). Strikingly, we also identified the presence of human α-syn deposits which appeared throughout the young A30P liver, localizing to regions near the portal and central veins (Fig. 2A–C). In some instances, we also observed the appearance of rosette-like α-syn deposits within the liver parenchyma (Fig. 2D–F). Interestingly, we also detected the presence of human α-syn deposits within the capsule of Glisson, the connective tissue of the liver which contains the vessels (data not shown). In aged A30P livers (18 months), we observed a progressive deposition of α-syn within the portal and central veins (Fig. 2G–I). We could also detect a significant increase in α-syn deposition within the liver parenchyma compared to young livers (Fig. 2J–L, Additional file 3: Fig. 3C). No staining or cross reactivity with mouse α-syn could be detected in neither A30P nor WT mice, regardless of age, when the primary antibody (14H) was omitted as a control experiment (Additional file 4: Fig. 4A–F). Notably, the aggressive accumulation of α-syn within the A30P liver appeared to correlate with the accumulation of α-syn within the brain of this PD model [34]. To eliminate the possibility that α-syn accumulation in the liver could be due to endogenous expression of the *SNCA* gene driven by the Thy-1 promoter, we performed semi-quantitative gene expression analysis (qRT-PCR) on Tg brain and liver samples as well as non-Tg tissue sample controls (18 months). Using specific probes targeting human α-syn (Additional file 10: Table I), we observed the expression of human α-syn in the brain of the A30P model but absent in WT mice. Moreover, neither A30P nor WT livers displayed any human *SNCA* mRNA expression (Additional file 4: Fig. 4G). We also assessed for the presence of endogenous mouse α-syn and as expected, we found such endogenous expression in the brain of WT and A30P mice but not in the liver (Additional file 4: Fig. 4H). Taken together, these findings show that α-syn in the liver in this mouse model likely originates from the brain. This could potentially be a way to facilitate clearance and detoxification out of the brain. Thus, our results suggest a potential liver involvement in the clearance of α-syn pathology.

### Characterization of human α-syn within the liver of A30P mice

**Fig. 3:**
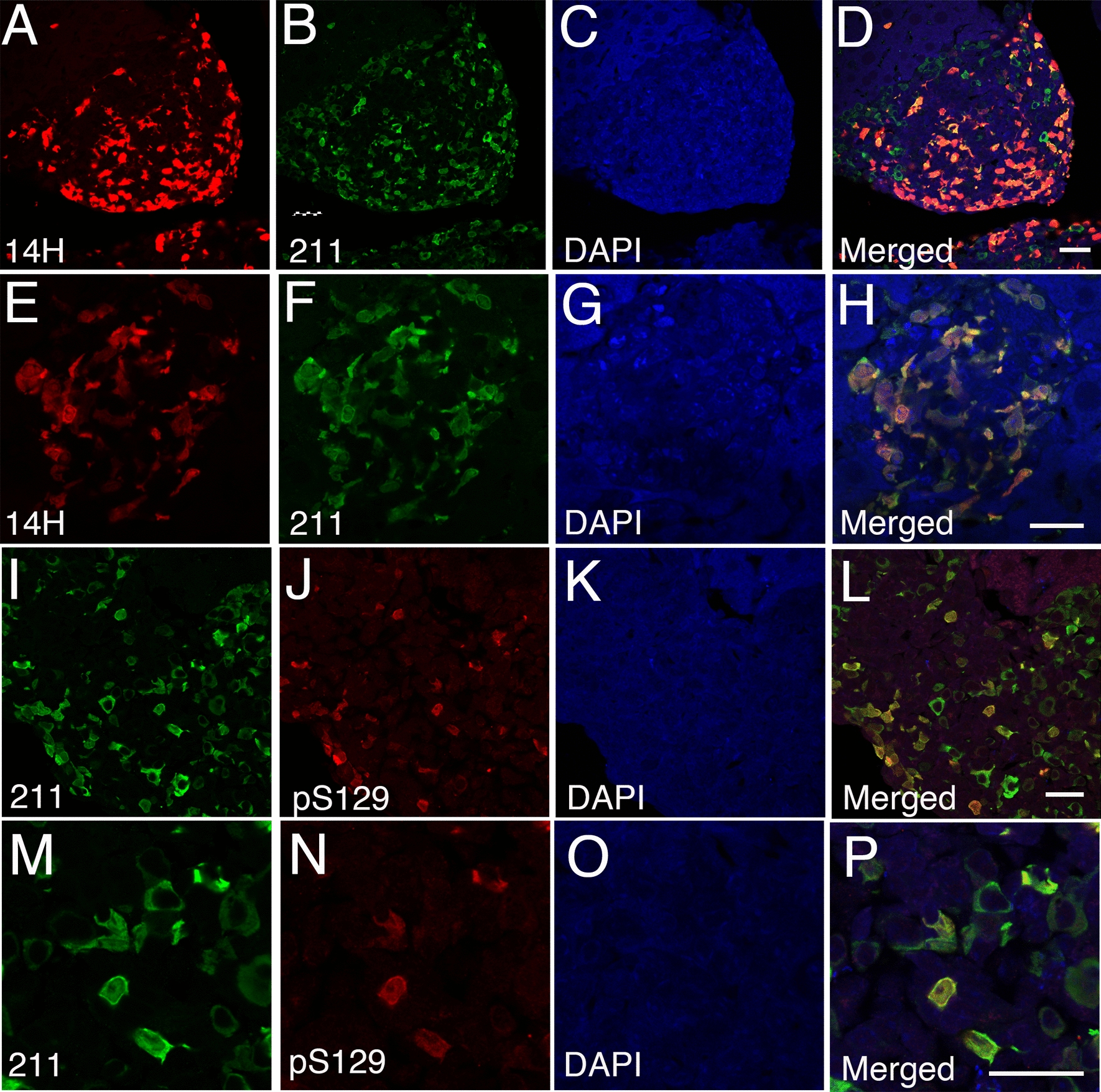
Human α-syn deposits within the A30P mouse liver are selectively phosphorylated at serine 129 (pS129). **A–H** Human α-syn deposits within the transgenic A30P mouse liver immunolabeled with the 14H antibody co-localize with the human specific 211 α-syn antibody. **I–P** Human α-syn deposits within the A30P liver are partially phosphorylated at serine 129. All tissue sections were counterstained with DAPI (blue). Bars = 20 µm

To further characterize the identity and accumulation of human α-syn within the liver of the A30P model, we performed immunohistochemistry on aged liver tissue sections using the widely used human specific α-syn clone Syn-211 (211) antibody which recognizes α-syn pathology in PD brain [53]. Using confocal image analysis, we observed a clear co-localization between the 14H and 211 antibodies, validating the identity of human α-syn within the liver of A30P mice (Fig. 3A–H). We next investigated whether the presence of α-syn within the aged liver is phosphorylated at serine 129 (pS129), a pathological hallmark associated with PD [54]. Using confocal image analysis, we observed a partial co-localization between pS129 and 211 antibodies (Fig. 3I–P), suggesting that the accumulation of α-syn pathology within the liver may undergo a similar post-translational modification to that in the brain. Alternatively, it is possible that human α-syn is transported from the brain to the liver in an already phosphorylated state.

**Fig. 4:**
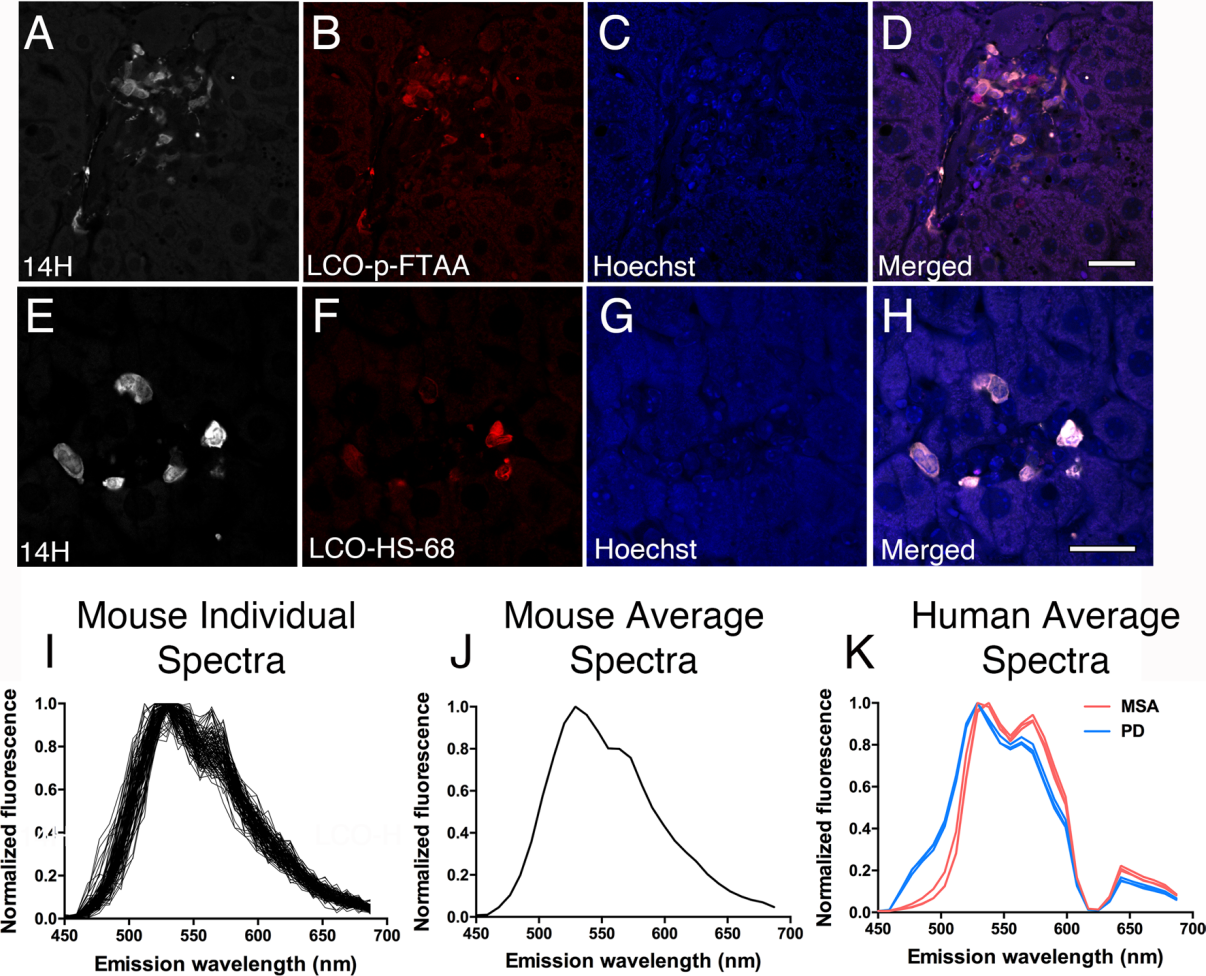
Human α-syn inclusions within the mouse A30P liver co-localize with luminescent conjugated oligothiophenes (LCOs). **A**–**D** Human α-syn deposits immunolabeled with the 14H antibody partially co-localize with the p-FTAA and **E–H** HS-68 LCOs in aged A30P livers. All tissue sections were counterstained with Hoechst (blue). **I** Individual and **J** average emission spectra obtained from HS-68-positive human α-syn deposits in the aged A30P liver. **K** Average emission spectra obtained from HS-68-positive α-syn aggregates from human PD brains (*n* = 3, blue) and human MSA brains (*n* = 3, red). Note that the spectra in the human brains were obtained using a filter which cuts off the emitted light at 625 nm in wavelength, which was not used for this study. **K** Adapted from [37] with permission. © The Author(s). 2019 Open Access. Creative Commons Attribution 4.0 International License (http://creativecommons.org/licenses/by/4.0/). Bars = 20 µm

We next investigated the aggregation state of α-syn within the liver using Thioflavin T, a molecular probe known to stain fully mature amyloid structures in multiple neurological conditions. In our hands, however, Thioflavin T failed to label any of the α-syn deposits within aged A30P liver (data not shown), suggesting a limited sensitivity of the probe and/or the lack of fully mature α-syn amyloidogenic structures within the liver. To overcome this technical limitation, we next stained tissue sections with a highly sensitive class of amyloid dyes commonly known as luminescent conjugated oligothiophenes (LCOs), which are ligands identified to stain protein aggregates that are formed early in the aggregation process [38–40, 55]. Using two well-characterized LCOs (p-FTAA and HS-68), we revealed the presence of p-FTAA-positive deposits (Fig. 4A–D) as well as a select number of HS-68-positive structures which co-localized extensively with the human specific α-syn antibody, 14H (Fig. 4E–H). These findings provide evidence for the presence of human α-syn within the liver in a partially aggregated state.

Given that the emission spectra of LCOs provide clues into the amylogenic state of its target [55], we next measured the wavelength spectra of α-syn liver deposits using the HS-68 ligand. Consistent with the lack of Thioflavin T staining, the emission spectra observed using the HS-68 LCO revealed two specific peaks, one peak around 530 nm and another peak approximately at 570 nm wavelength (Fig. 4I, J). A similar spectral signature was previously reported for immature amyloid beta (Aß) and tau aggregates in brains of young transgenic AD mice and might therefore be indicative of less mature assemblies [55, 56]. Interestingly, the emission peaks of the HS-68-positive α-syn deposits within the A30P liver resembled the peaks previously obtained from human PD brains using the same ligand but were markedly different to the wavelengths observed for MSA brain deposits (compare Fig. 4J with K [37]). Collectively, our results indicate that the A30P liver is susceptible to α-syn accumulation, thus suggesting a potential liver involvement in the clearance and detoxification of pathological protein aggregates from the brain.

### Striatal injection of α-syn assemblies localize to the liver in WT mice

**Fig. 5:**
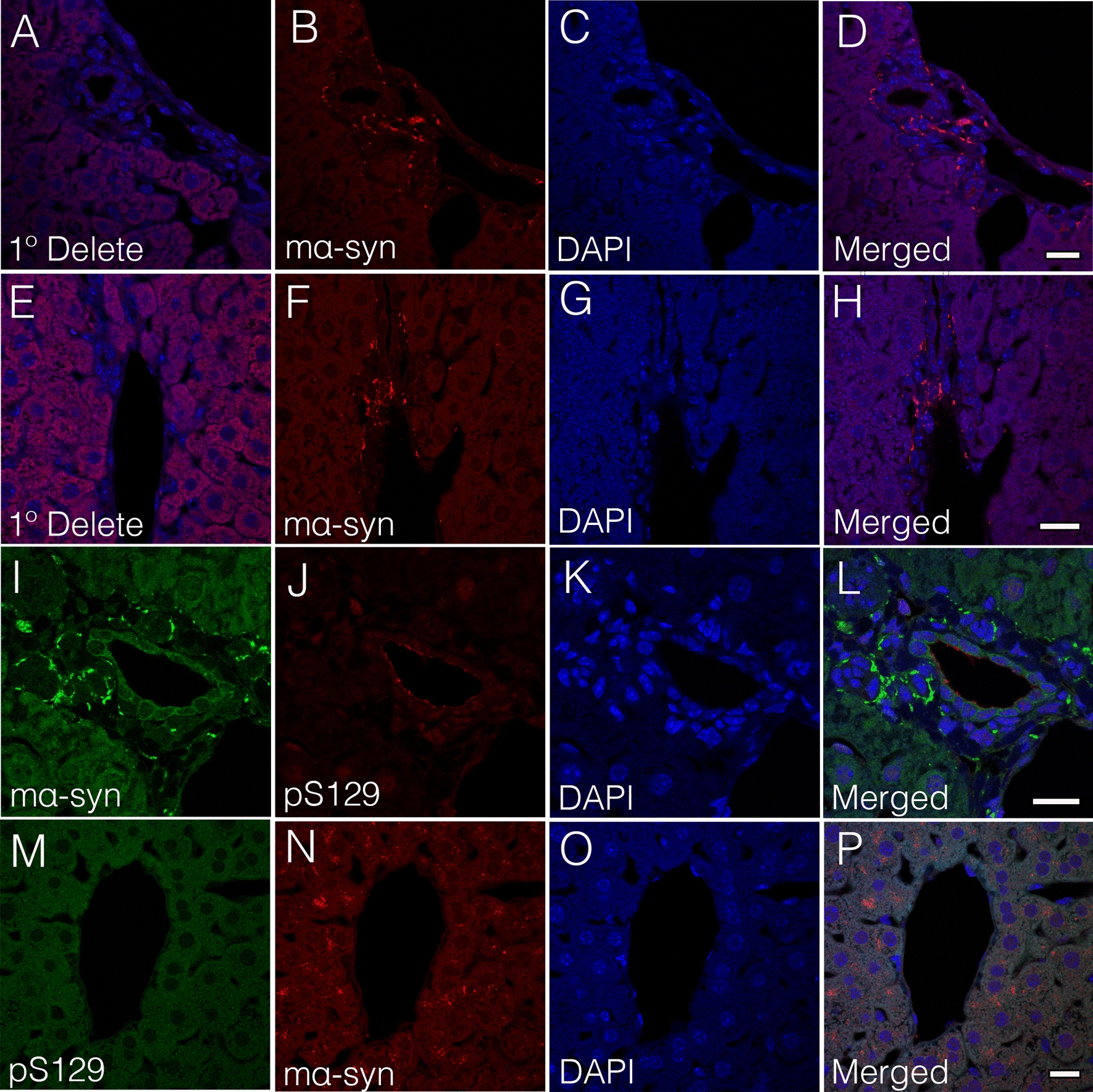
Alpha-synuclein is transported from the brain to the liver following a single striatal injection of mouse α-syn (mα-syn) oligomers. **B**–**D, F–H** mα-syn oligomers are detected within the portal tracts of the wild type mice 1-month post striatal injection. **A,** **E** Adjacent tissue sections lacking primary antibody (1°delete) show no mα-syn immunoreactivity. **I–L** Injected mα-syn oligomers detected within the portal tracts or **M–P** liver parenchyma are not phosphorylated at serine 129 (pS129) 1-month post injection. All tissue sections were counterstained with DAPI (blue). Bars = 20 µm

To further explore whether α-syn is transported from the brain to the liver, we took advantage of a widely established mouse model of PD involving α-syn injections in the brain leading to α-syn self-assembly and accumulation that is detected within the brain approximately within 20–30 days post-protein injection [57, 58]. Thus, we performed a single striatal injection of mouse oα-syn assemblies into wild type mice and assessed their presence within the liver 30 days post injection. Consistent with the detection of human α-syn within the A30P liver, we observed the appearance of injected oα-syn assemblies within the portal tracts (Fig. 5B–D), liver parenchyma and sinusoidal regions, the specialized channels which allow blood flow from portal tracts to the hepatic venule (Fig. 5F–H). As expected, adjacent control sections not treated with α-syn primary antibody showed no α-syn staining (Fig. 5A, E). Similarly, and consistent with our qRT-PCR analysis, we observed no staining within the portal tracts or liver parenchyma in non-α-syn injected wild type mice (data not shown). We then assessed whether the presence of injected oα-syn assemblies within the liver were pS129 positive. In contrast to the aged liver tissue sections from the A30P mouse model however, we found no evidence of pS129 immunoreactivity within either the portal tracts (Fig. 5I–L) or hepatocytic structures 1-month post-protein injection (Fig. 5M–P). These results suggest that α-syn is transported from the brain to the liver, likely via the circulatory system. Alternatively, it is also possible that the vagal or splanchnic nerves which directly innervate the liver via the portal area may play role in this process.

### Human α-syn accumulation within the liver promotes inflammation

**Fig. 6:**
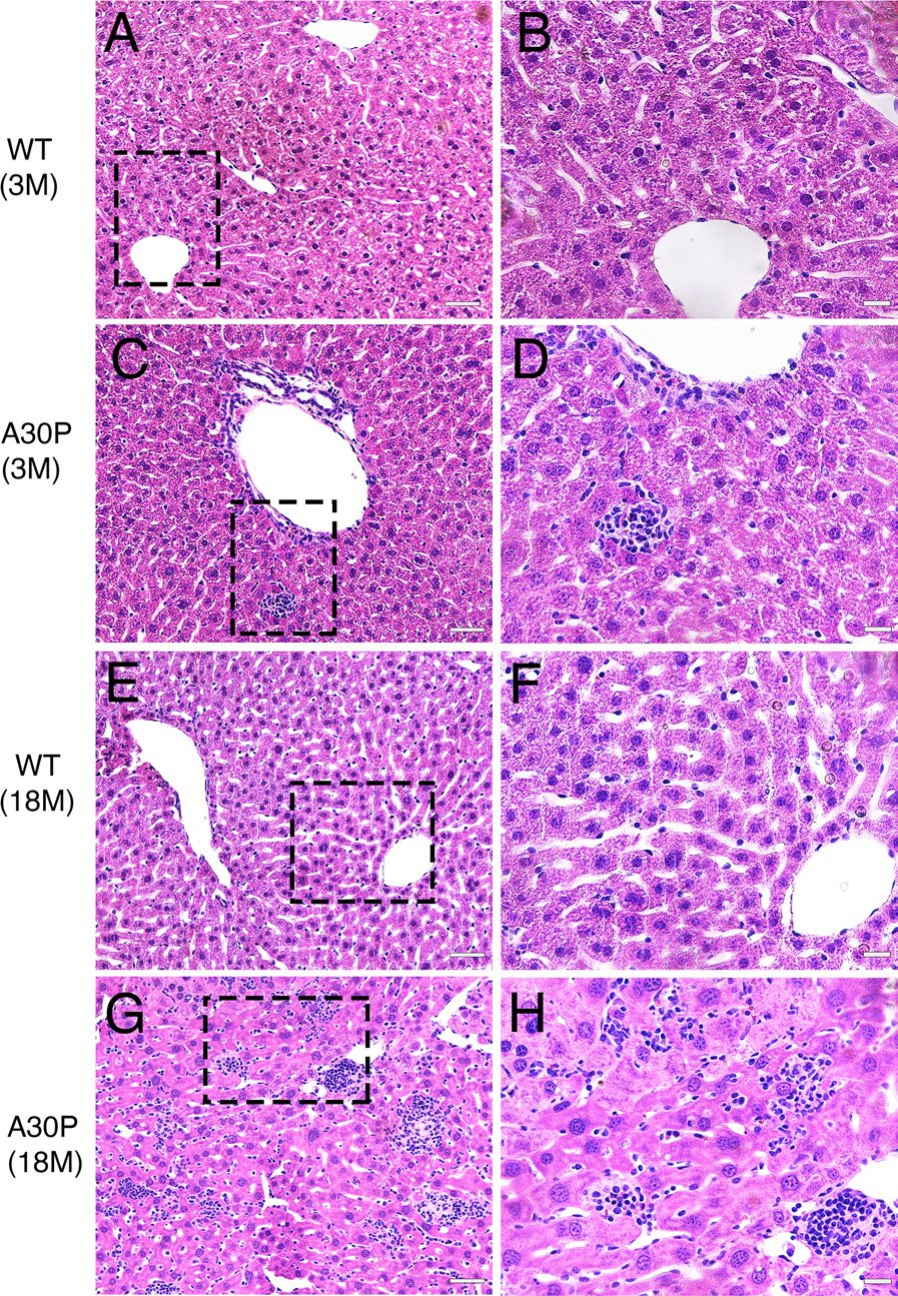
The A30P mouse model of PD shows a progressive inflammation within the liver. **A**, **B** Hematoxylin and Eosin (H&E) staining shows no detectable inflammation within the liver in young (3 months) or **E, F** aged (18 months) wild type mice. **C, D** H&E staining within the young A30P liver shows focal inflammation within the liver parenchyma that is progressive and much more prominent in the aged A30P liver (**G, H**). Bars = 20 µm

Given the inflammatory response generated by α-syn deposition in the brain and other organs outside the CNS, we next investigated whether the A30P liver is susceptible to inflammation as a result of a progressive α-syn deposition. We therefore performed hematoxylin and eosin (H&E) staining on young (3 months) and aged (18 months) liver tissue sections from WT and A30P mice. In WT mice, we observed no clear signs of inflammation regardless of age (Fig. 6A, B, E, F). In contrast, the A30P liver showed a progressive inflammatory pattern that appeared already by 3 months of age (Fig. 6C, D). Indeed, we observed focal inflammation areas throughout the liver parenchyma (Fig. 6C, D, squares). At 18 months of age, we observed a much more severe inflammatory reaction with more extensive focal inflammation within the liver parenchyma as well as in the portal tracts (Fig. 6G, H). In these livers, however, we found no evidence of fibrosis or steatosis in either WT or A30P mice regardless of age (Additional file 12: Table III). These findings suggest that the progressive accumulation of α-syn deposits within the A30P liver may be directly responsible for the progressive inflammation observed, as no detectable signs of inflammation were observed in WT mice regardless of age. These findings are consistent with the α-syn aggregation-dependent inflammation observed in the brains of the A30P mice and in PD patients [34].

### Human α-syn within the liver partially co-localizes with inflammatory markers

**Fig. 7:**
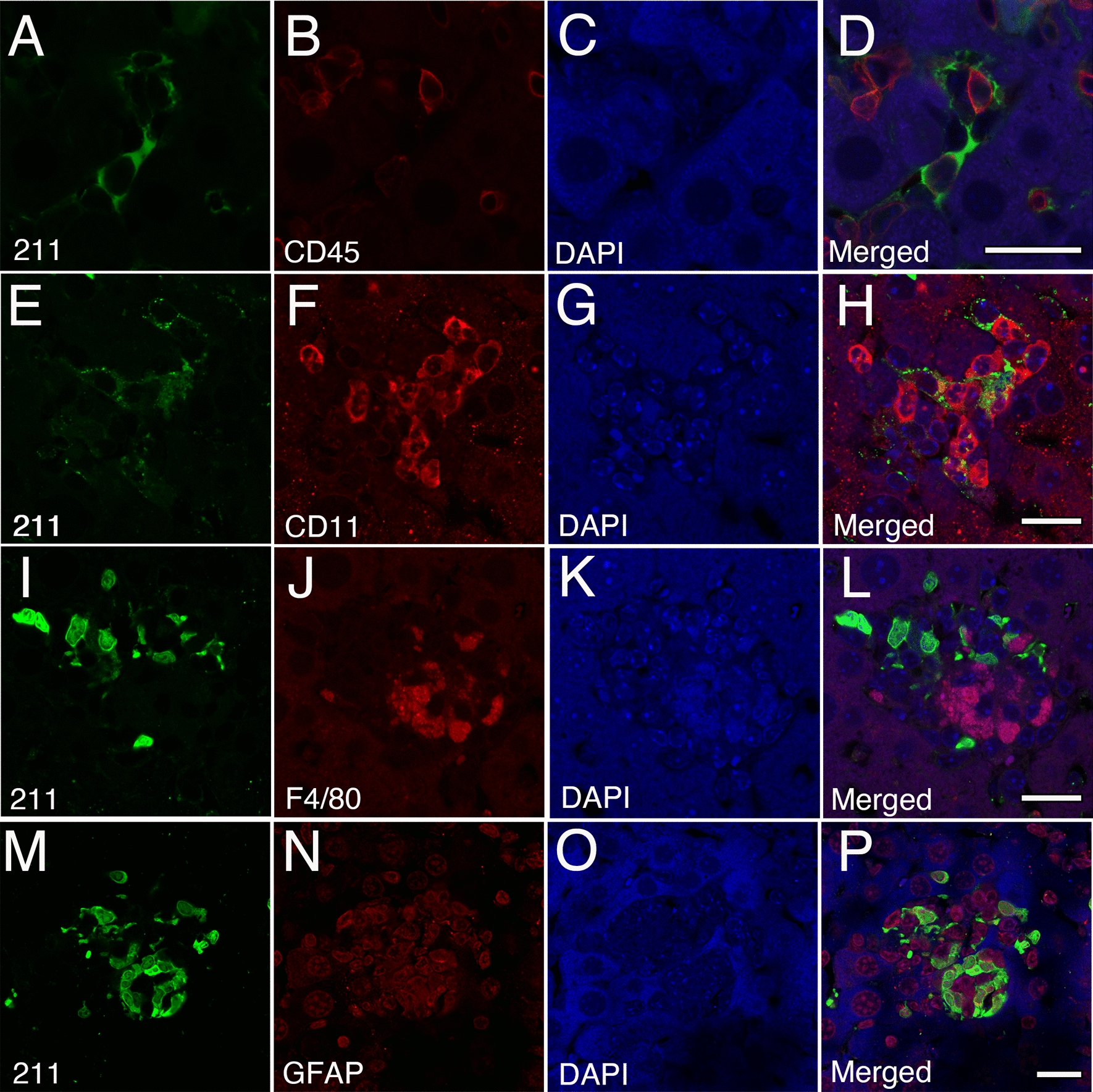
Accumulation of human α-syn within the liver promotes inflammation in the A30P mouse model of PD. **A**–**D** Confocal image analysis on aged A30P liver sections demonstrate the presence of CD45-positve leukocytes, **E**–**H** CD11b-positive monocytes and **I**–**L** F4/80-positive Kupffer cells next to or in close proximity to α-syn deposits. **M**–**P** Partial co-localization between α-syn and the GFAP-positive perisinusoidal cells within the sinusoidal region. All tissue sections were counterstained with DAPI (blue). Bars = 20 µm

Given the progressive inflammatory state of the A30P liver compared to livers from wild type mice, we next assessed whether inflammatory cells co-localize with α-syn following its transport to the liver. Thus, we performed confocal image analyses on tissue sections from aged A30P mice and identified the presence of CD45-positve leukocytes (Fig. 7A–D), CD11b-positive monocytes (Fig. 7E–H), and F4/80-positive Kupffer cells (Fig. 7I–L) that were next to or in close proximity to human α-syn deposits. However, none of these cell types co-localized with α-syn. Similarly, immunohistochemical staining for CD3 or MPO (myeloperoxidase) revealed the presence of T-cells and granulocytes within the liver parenchyma respectively, however, these cell types were also devoid of any human α-syn as no co-localization was observed (data not shown). Interestingly, we did observe a partial co-localization with the GFAP-positive perisinusoidal cells commonly known as hepatic stellate cells (Fig. 7M–P), specialized liver cells involved in fibrosis following liver injury [59], thus suggesting that in addition to inflammation, α-syn may promote liver toxicity. Taken together, our observations suggest that human α-syn is likely transported from the brain to the liver and its progressive accumulation within the liver promotes cellular toxicity resulting in liver inflammation.

### Accumulation of α-syn within the liver is a general phenomenon of synucleinopathies

To further determine whether the accumulation of α-syn in the liver is not specific to the A30P model but rather a general phenomenon of synucleinopathies, we investigated the presence of human α-syn within the liver in a model of synucleinopathy overexpressing normal WT α-syn also under control of the Thy-1 promoter (L61, Additional file 11: Table II) [35]. As expected, we observed a progressive accumulation of human α-syn in the brain of this model between 3 and 12 months of age (Additional file 5: Fig. 5A and H, inserts). Consistent with our results from the A30P model, we also observed the presence of human α-syn within the liver of young L61 mice (3-month old) as small puncta distributed focally in portal veins and liver parenchyma (Additional file 5: Fig. 5A–F). In aged L61 livers 12 months old, we could reveal a progressive accumulation of human α-syn deposits (Additional file 5: Fig. 5G–L). Notably, α-syn deposition within the L61 liver appeared to occur to a much lesser degree than for the A30P model at any given time point analyzed (Additional file 12: Table III), despite the expression of both proteins under control of the same neuronal promoter (Additional file 11: Table II). Thus, the accumulation of liver α-syn appears to correlate with the amount of α-syn deposition within the brain of these synucleinopathy models [60].

We next assessed for the presence of α-syn within the liver of transgenic mice modeling MSA. These animals express human α-syn within oligodendrocytes approximately 3-times the levels human α-syn (Additional file 11: Table II) under control of the myelin basic promoter (MBP, MBP29) [36, 45]. At 4 months of age and consistent with the PD models above, we observed the presence of human α-syn deposits throughout the MBP29 brain (Additional file 6: Fig. 6A, insert). Within the MBP29 liver, we observed occasional α-syn puncta that were randomly distributed throughout the liver parenchyma (Additional file 6: Fig. 6A–C). In some instances, we also observed the presence of human α-syn surrounding the inflammatory cells within the sinusoidal region (Additional file 6: Fig. 6D–F). In contrast to the A30P model, α-syn pathology within the MSA mouse liver at 4 months was negative for pS129 immunostaining (data not shown). As expected, tissue sections lacking primary antibody were devoid of any human α-syn staining (Additional file 6: Fig. 6G–I). To also eliminate the possibility that human α-syn accumulation within the MBP29 liver is due to expression of the human *SNCA* gene, we performed qRT-PCR on brain and liver tissue samples from MBP29 and WT control mice. Using specific probes targeting human α-syn (Additional file 10: Table I), we observed restricted expression of the human *SNCA* transgene to the brain of this mouse model and as expected, absent in both the MBP29 and WT liver samples (Additional file 7: Fig. 7). Importantly, the limited lifespan of this aggressive MSA model [36] prevented us from investigating the accumulation of human α-syn pathology in older mice.

Finally, we assessed whether other proteins associated with neurodegenerative disorders are also deposited within the liver. To this end we performed immunohistochemistry on brain tissue sections from the samples from mice expressing the human amyloid precursor protein (APP) harboring the Beyreuther/Iberian mutation (*App*^*NL−F*^ mice) thus modeling AD [61]. As expected, we observed a clear accumulation of human amyloid beta (Aβ) within the brain of this mouse model at 24 months of age (Additional file 8: Fig. 8A, insert). However, and in contrast to the progressive α-syn deposition observed within the synucleiopathy mouse models shown above (A30P and L61), the *App*^*NL−F*^ mice (20 months) lacked any detectable age-dependent Aβ accumulation within the liver (data not shown). In mice of advanced age, however (24 months), we observed the presence of rare Aβ inclusions within the liver positive for an array of widely used human specific Aβ antibodies (Additional file 8: Fig. 8A–F). As expected, the lack of primary antibodies or immunostaining on WT mice failed to detect any Aβ inclusions (Additional file 8: Fig. 8G–L). Moreover, H&E analysis revealed no signs of inflammation in the liver even in the overly aged animals, thus validating the lack of local Aβ accumulation in this mouse model of AD (data not shown). Taken together, our immunohistochemical observations revealed a progressive accumulation of α-syn within the liver of synucleinopathy models that is absent in a model of AD. Intriguingly, accumulation of α-syn was more extensive in the A30P PD model [4] followed by L61 [2] and lastly the MSA model [1] (Additional file 12: Table III). Collectively, these findings suggest that the propensity for α-syn aggregation within the brain and the aggregation state play a role in its accumulation in the periphery, supporting the idea that α-syn within the liver in these models may originate from the brain.

### Identification and characterization of α-syn pathology within the liver in PD cases

**Fig. 8:**
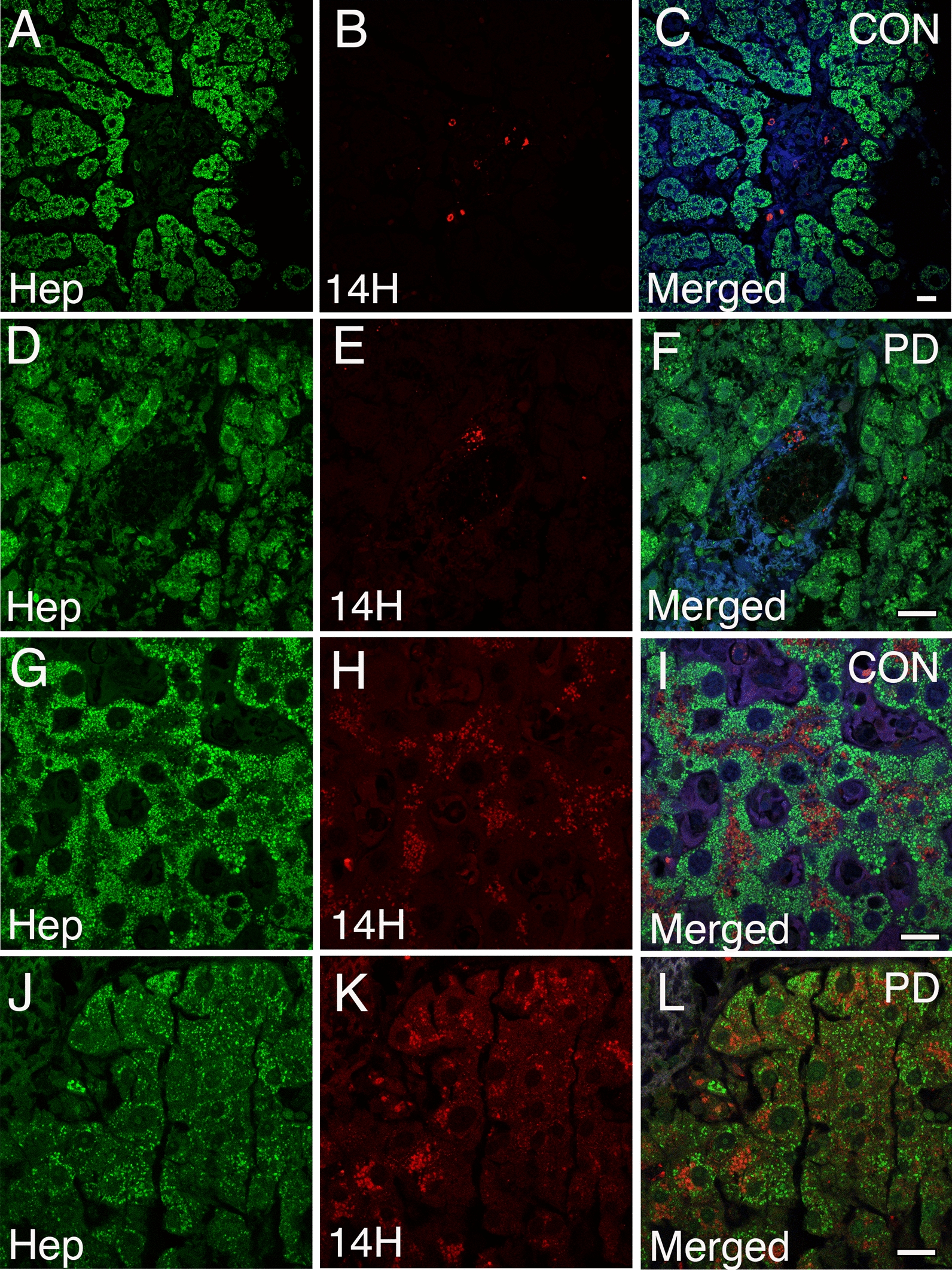
Accumulation of human α-syn within the human liver. **A**–**C** Confocal image analysis on human liver tissue sections immunolabeled with the 14H human-specific antibody (red) and anti-hepatocyte antibody (Hep, green) demonstrates the presence of human α-syn deposits within the sinusoidal regions, **D**–**F** portal tracts, and liver parenchyma (**G**–**L**) in PD and age-matched controls. Note that α-syn within liver hepatocytes appear as round puncta and often near nuclei. Importantly, not all hepatocytes within the affected region contain α-syn deposition. All tissue sections were counterstained with DAPI (blue). Bars = 20 µm

To validate the accumulation of α-syn within the liver as a pathological phenomenon in PD we assessed Lewy Body Disease cases (LBD, *n* = 16) with confirmed α-syn deposition in the brain (Braak 5–6, Additional file 13: Table IV) as well as aged-matched controls with no evidence α-syn deposition in the brain (M = 79.8 vs 76.5, *n* = 14). In a double-blinded setting, we identified the presence of α-syn within the liver that was detected by human-specific α-syn antibodies in several neuropathologically confirmed cases as well as in age-matched control tissues (Additional file 13: Table IV). Similar to our mouse models of PD, the presence of α-syn within the human liver was located to the sinusoidal regions, portal tracts, as well as liver parenchyma (Fig. 8A–L). Moreover, α-syn accumulation within hepatocytes appeared random but most often as round puncta near the nuclei (Fig. 8G–L). Importantly, even within the same affected region, not all hepatocytes contained α-syn and therefore did not seem to be equally vulnerable to α-syn deposition (Fig. 8G–L). Overall, we identified α-syn pathology within the liver in 12 out of 16 neuropathologically confirmed cases with α-syn pathology in the brain (75%). Out of these 12 cases, 6 showed α-syn accumulation within hepatocellular structures (37.5%). Similarly, 8 out of 14 control cases were positive for liver α-syn pathology (57%), whereas 4 of them showed α-syn within hepatocytes (28%). Only occasional cases showed detectable evidence of fibrosis, steatosis or inflammation but this did not correlate with brain pathology or α-syn accumulation within the liver (Additional file 13: Table IV). However, cholestasis was detected in 14 out of 16 neuropathologically confirmed cases (87.5%), whereas 9 out of 14 controls with no α-syn deposition in the brain were positive for the same condition (64%) (Additional file 13: Table IV). We then assessed whether PD or control cases positive for α-syn within the liver show pathological phosphorylation at serine 129 (pS129). While α-syn deposition within the liver was validated with the human-specific 211 antibody, none of the cases analyzed contained pS129 immunoreactivity (Additional file 9: Fig. 9A–L). Collectively, our data demonstrates that α-syn accumulation occurs in aged human livers with a tendency for higher prevalence in neuropathologically confirmed cases with α-syn deposition in the brain (75%) relative to controls without α-syn accumulation (57%). Thus, the propensity for α-syn accumulation within liver tissue may be indicative of a liver’s role in pathological protein clearance derived from either the brain or peripheral tissues.

## Discussion

Parkinson’s disease is a neurodegenerative disease with a broad spectrum of motor and non-motor manifestations. Accumulating evidence now demonstrates that the distribution of α-syn pathology is not limited to the brain but also extends to the peripheral autonomic neuronal network [9, 62]. Indeed, α-syn protein deposition has been identified in multiple organs outside the CNS in patients with PD (reviewed in [9]). Similarly, several studies have previously demonstrated gastrointestinal and peripheral organ dysfunction in transgenic animals modeling PD by overexpression of human α-syn under a CNS-specific promoter. For instance, the L61 mouse model has been reported to have severe distention and large intestine blockade, symptoms indicative of constipation. Morphologically and compared to littermate controls, the L61 model appears to develop swollen and pallor intestines as well as large urinary bladders, particularly in aged mice (12–15 months) [63]. Moreover, these mice have been demonstrated to have alterations in the colonic myenteric ganglia thus showing deficits in defecation [64]. Although similar studies have not been performed in the A30P model, Kuo and colleagues demonstrated that mice expressing the A30P or the A53T mutation driven by the P1 artificial chromosome (PAC) robustly show enteric nervous abnormalities. Importantly, while these mice (A30P or A53T) show abnormal motor behavior, neither model demonstrate olfactory deficits nor α-syn inclusions, the pathological hallmarks of PD. Nonetheless, they recapitulate the early gastrointestinal abnormalities observed in PD patients prior to neurodegeneration [65].

Consistent with the accumulation of α-syn in peripheral tissues in PD, we now report a putative liver involvement in the disease progression and demonstrate for the first time that human hepatocytes can take up oα-syn assemblies in vitro. The ability of human hepatocytes to take up α-syn from the extracellular milieu was validated using two hepatocyte cellular models consisting of primary human hepatocytes and hepatocytes derived from the human HuH-7 cell line [43]. Consistent with previous results [31], we demonstrate a protein–protein interaction between oα-syn and Cx32 in human hepatocytes, the main cell type within the liver and the main organ responsible for substance clearance and detoxification in the body [33]. Thus, providing further evidence for Cx32 in the selective uptake of oα-syn assemblies and pinpointing a potential liver involvement in the clearance of pathological protein aggregates either from the brain or peripheral tissues, such as the gut. It is worth noting that human hepatocytes lacking Cx32 can also take up oα-syn from the extracellular milieu, albeit to a lesser extent than hepatocytes expressing Cx32. These data thus suggest that other putative receptors/binding partners present within hepatocytes (or other cell types within the liver) also may play a role in this process. The identity of these putative receptors, however, remains to be further investigated.

In vivo, we demonstrate the presence and progressive accumulation of α-syn deposits within the liver in animal models of PD (A30P, L61). Although α-syn protein expression in neurons is under control of the same neuronal promoter in both A30P and L61 mice (Thy-1), the distinct degrees of α-syn liver accumulation may be due either to the different forms of human α-syn expressed (A30P vs wild-type) or to the differences in transgenic copies between these models. Thus, suggesting that α-syn deposition within the liver may be dependent on the brain region expressed, protein expression levels, the amount of α-syn deposits in the brain and/or the susceptibility of aggregation of the molecule (mutant vs WT), as well as the cell type in which α-syn is expressed (neurons vs oligodendrocytes) [60]. In contrast to our synucleinopathy models, the amyloid precursor protein (APP) knock-in mice modeling AD used in this study only showed rare Aβ inclusions that could only be detected in quite old mice. Our results thus suggest liver involvement in the clearance of α-syn pathology and that brain to liver transmission may be an additional feature of synucleinopathies.

Currently five *SNCA* mutations have been described to cause early onset forms of PD (A30P, E46K, H50Q, G51D, and A53E/G/T/V) [66, 67]. While A53T, E46K and H50Q promote higher rates of α-syn aggregation, the A30P mutation appears to cause a slower fibrillar formation rate which thereby promotes oligomer formation [66]. Similarly, the G51D mutation has been reported to decrease α-syn aggregation but patients with this mutation have a much earlier disease onset and accumulation of α-syn within oligodendrocytes, similar to MSA [68, 69]. It will be interesting to determine whether higher aggregation-dependent mutants in other animal models of PD demonstrate a higher degree of pathology within the liver or peripheral organs compared to the transgenic models reported in this study. Nonetheless, we further demonstrate that a selected number of α-syn structures within the aged A30P liver are partially phosphorylated at serine 129 (pS129-positve), suggesting that these α-syn aggregates may be pathological in nature as shown by the progressive inflammation state of the liver using H&E. Moreover, a number of α-syn structures were also positive for the LCOs p-FTAA and HS-68, with a spectral signature indicating partly mature aggregates, but lacking the fully amyloidogenic properties identified by Thioflavin T. It is also worth noting that the LCO spectrum of the A30P liver α-syn inclusions was similar to what was earlier observed from α-syn aggregates in PD brain tissue [37]. However, in that study ethanol or acetone fixed fresh-frozen human brain sections were investigated, while the current study used formalin fixed paraffin-embedded tissue samples, which might have an effect on the LCO spectral properties. Nonetheless, it remains to be determined whether α-syn is transported in an aggregated/modified state or is progressively modified within the liver as it is in the brain.

While we corroborated brain to liver transport following a single stereotactic injection of α-syn oligomers into the striatum of wild type mice 1-month post-protein injection, the route of delivery from the brain to liver remains unknown for both mice and humans. It is possible that the nodosa ganglia efferent neurons which connect the portal veins and hepatic arteries play a role in this process. Indeed, branches of both the vagal and splanchnic nerves directly innervate the liver via the portal area, and these connections are associated with the portal vein and bile ducts (for review please see [70]). Furthermore, multiple studies have shown the presence and progressive accumulation of α-syn within the vagal nerve leading to the dorsal motor nucleus of the vague following injections in the gut [27]. Alternatively, it is possible that an excess of pathological α-syn in the brain is secreted into the blood stream during PD progression and is exported to the liver via the circulatory system. Indeed, the red blood cells have been identified to carry α-syn in the blood stream [71]. Moreover, the blood–brain barrier (BBB) has been demonstrated to play a role in the delivery of α-syn to multiple organs [72]. Sui and colleagues demonstrated that radioactively labeled α-syn can successfully cross the BBB in both the brain-to-blood and blood-to-brain directions, suggesting a potential circulatory involvement in the transport of α-syn out of the brain [72]. Alternatively, it is possible that the presence of α-syn pathology in the gut plays a role in this process since blood from the gut (a region known to harbor α-syn pathology in PD) passes through the liver, where toxic α-syn assemblies can be filtered out before further transfer to brain and other organs. However, the neuropathologically confirmed cases or controls used in this study were not pathologically assessed for the presence of α-syn in the gut or other peripheral organs.

Although we could identify α-syn pathology in the human liver, we were not able to pinpoint any significant differences on the location, accumulation (fluorescence intensity) or cellular morphology between neuropathologically confirmed cases and controls. Moreover, we could not explain why certain clinically diagnosed PD liver cases with high α-syn burden in the brain (Braak 5–6, 2 out of 16) had no detectable α-syn pathology in the liver. However, we cannot rule out the presence of α-syn pathology in other parts of the liver, given the size of the human liver and the random distribution of α-syn pathology. On the other hand, we also observed aged-matched control cases without evidence of neurodegeneration but with α-syn pathology in the liver (8 out of 14). The discrepancy to the findings in the animal models might be explained by the pure, brain-only expression of the α-syn driven by CNS-specific promoters in these models. While in the human cases α-syn could potentially reach the liver both from the brain and the periphery in line with bottom-up transmission hypothesis which indicates that the enteric/gut region is first affected with α-syn pathology, before the pathology progresses to the brain [28]. However, as stated above, none of the cases presented in this study were assessed for α-syn accumulation outside the CNS. Thus, additional studies are needed to further validate a gut to liver α-syn transmission in PD patients without α-syn deposition in the brain [28]. Moreover, based on the frequency of liver α-syn accumulation in non-LBD cases identified in this study it suggests that it is possible that α-syn can be found in the liver also in individuals that may not be on the path to disease development. Thus, further studies are needed to fully understand the source of α-syn within the liver. Together with the findings from the animal experiments it is reasonable to hypothesize that in LBD-cases, liver α-syn accumulation could in part, originate from the brain.

It is worth noting that over a decade ago, Ltic and colleagues reported the presence of multiple endogenous protein α-syn isoforms within the liver, kidney, lung, heart, adrenal gland and testis of both fetal and adult rat as well as human tissue samples [73]. The identified α-syn isoforms appeared as three different bands of different molecular weights identified by Western blot analysis (19, 36 and 52 kDa in size) [73]. However, using qRT-PCR analysis we did not identify any endogenous or human α-syn expression within the liver, thus the accumulation of α-syn in the mouse liver likely indicates a brain or peripheral delivery. In humans, Ltic and colleagues reported a decrease in the presence of α-syn within peripheral tissues which correlated with cellular maturation, thus showing no expression of α-syn in adult liver samples [73]. Although qRT-PCR was not performed on human liver tissue in the present study to assess for α-syn expression, these findings support that the presence of α-syn identified in adult human liver may be abnormal thus further indicating that the liver plays a role in clearance of PD related pathology.

Taken together, while multiple organs outside the CNS in PD cases have been reported to contain α-syn deposits, our study is the first to identify the presence of α-syn within liver hepatocytes in both cellular and animal models of PD as well as in humans. While we could identify α-syn deposition both in PD cases and non-diseased control subjects, the pathology was more common in PD patients. Together with our findings from the cell and animal experiments it is reasonable to conclude that liver α-syn could originate from the brain in the LBD-cases. Although the role of α-syn deposition within the liver both in humans and mice remains unknown, we hypothesize that α-syn is delivered from either the gut/peripheral tissues or brain to the liver before being cleared out of the body, as part of the organ’s detoxification and clearance process. Indeed, we showed that human hepatocytes are capable of lowering the levels of α-syn over time. Moreover, the increase in the inflammatory state observed in aged A30P mouse model suggests that if the capacity of pathological protein clearance is insufficient, accumulated α-syn can lead to liver toxicity. In conclusion, we hypothesize that the liver is involved in the clearance of pathological protein aggregates, which may be a crucial mechanism in the removal of neurotoxic α-syn in PD and related synucleinopathies.

## Supplementary Information

**Additional file 1: Figure 1.** Primary human hepatocytes take up oligomeric α-syn assemblies in vitro. A–F) Confocal image analysis of primary hepatocytes incubated with ATTO-550 labeled oa-syn (red) and then immunolabeled with Cx32 (green) demonstrate the internalization of oα-syn and a partial co-localization between the gap junction protein Cx32 (yellow, orthogonal views). All cells were counterstained with DAPI (blue). Bars = 10 μm.

**Additional file 2: Figure 2.** Immunoprecipitation of human α-syn oligomers pulls down Cx32. A–D) Immunolabeling on primary human hepatocytes or E–H) HuH-7 cells expressing Cx32 (red) using the human specific 14H antibody show no α-syn reactivity in the absence of α-syn treatment. I) Immunoprecipitation of human α-syn oligomers in HuH-7-Cx32 cells treated with α-syn oligomers pulls down Cx32 whereas untreated cells show no Cx32 pulldown and only the low and heavy chains of the antibody used are shown. J) Confocal image analysis of HuH-7 and HuH-Cx32 cells showing the morphology with or without Cx32 expression. Bars A–H = 20 μm and J= 50 μm.

**Additional file 3: Figure 3.** Cx32 expression in HuH-7 cells promote α-syn uptake. A, B) oα- syn uptake in WT HuH-7 cells (white bars) or HuH-7 cells expressing Cx32 (dotted bars) for different time periods using Western blot analysis. C) Quantification of human α-syn deposits in young (3 months) and aged (18 months) A30P liver tissue sections demonstrates a progressive accumulation α-syn over time. *p<0.05.

**Additional file 4: Figure 4.** Human and mouse α-syn expression is restricted to the brain. A–C) Confocal image analysis of liver tissue sections from aged A30P show no immunoreactivity to α-syn D–F) WT livers in the presence or absence of primary antibody (14H) show no immunoreactivity to mouse α-syn. G) qRT-PCR analysis of human α-syn in A30P brain, liver and normal wild type brains. H) qRT-PCR analysis of endogenous mα-syn in A30P brain, liver and normal wild type brain and liver. n.s.= non-significant. ****p<0.0001, Bars = 20 μm.

**Additional file 5: Figure 5.** Age dependent accumulation of human α-syn deposits within the liver of the L61 model of PD. A–F) Confocal image analysis using the 14H antibody shows the presence of human α-syn deposits (red) in young liver sections (3 months) as small puncta located within the portal tracts and liver parenchyma. Insert within panel (A) shows the deposition of α-syn within the brain of the L61 mice at 3 months of age immunostained with pS129 antibodies (green). G–L) Aged liver tissue sections (12 months) showing the progressive accumulation of human α-syn deposits within the portal tracts and liver parenchyma within the L61 model. Insert within panel (H) shows the deposition of α-syn at 12 months of age immunostained with pS129 antibodies (green). All tissue sections were counterstained with DAPI (blue). Bars = 20 μm.

**Additional file 6: Figure 6.** Identification of human α-syn deposits within the MBP29 liver modeling MSA. A–C) Confocal image analysis using the 14H antibody shows the presence of human α-syn deposits (red) in young liver sections (4 months) as small puncta located within the portal tracts and liver parenchyma. Insert within panel A shows the deposition of α-syn within the brain of the MBP29 mice at 4 months of age immunostained for total α-syn (green). D–F) In some instances, we identified the presence of human α-syn within the sinusoidal region likely surrounding inflammatory cells. G–I) Tissue sections lacking primary antibody (14H) show no human α-syn immunoreactivity. All tissue sections were counterstained with DAPI (blue). Bars = 20 μm.

**Additional file 7: Figure 7.** Human and mouse α-syn expression in MBP29 mice is restricted to the brain. A) qRT-PCR analysis of human α-syn in MBP29 brain, liver as well as non- transgenic (non-Tg) wild type brains and liver samples. n.s.= non-significant. ****p<0.0001.

**Additional file 8: Figure 8.** Identification of amyloid beta (Aß) deposits within the liver of NL-F mice liver modeling AD. A–C) Confocal image analysis of NL-F livers (24-month) using the antibody 6E10. Insert within panel A shows the deposition of α-syn within the brain of the NL-F mice at 24 months of age immune-stained with N82E antibodies (green). D–F) N82E shows the presence of human Aß inclusions (green) which co-localize with the rabbit monoclonal mOC65 antibody (red) within the liver parenchyma. G–I) Tissue sections lacking primary antibody (6E10, mOC64) show no Aß immunoreactivity. Bars = 20 μm.

**Additional file 9: Figure 9.** Accumulation of α-syn within the human liver lacks pS129 immunoreactivity. Confocal image analysis on human liver tissue sections immunolabeled with the 211 human-specific antibody (green) demonstrates the presence of human α-syn deposits within human hepatocytes, portal tracts, and liver parenchyma, however, negative for pS129 immunoreactivity both in PD (A–F) and aged-matched controls (CON) (G–L). Bars = 20 μm.

**Additional file 10: Table I.** Primary antibodies and primers used in this study.

**Additional file 11: Table II.** Description of animal models of neurodegeneration used in this study.

**Additional file 12: Table III.**  Liver characterization of animal models of PD and MSA.

**Additional file 13: Table IV.** Clinical and pathological characterization of human cases included in this study.

## Data Availability

All data is provided within this manuscript and supplemental materials.
