# Supplementary figures and images for: Accumulation of alpha-synuclein within the liver, potential role in the clearance of brain pathology associated with Parkinson’s disease
Source: Acta Neuropathol Commun. 2021 Mar 20;9:46. doi: 10.1186/s40478-021-01136-3 (PMC7980682; doi:10.1186/s40478-021-01136-3)

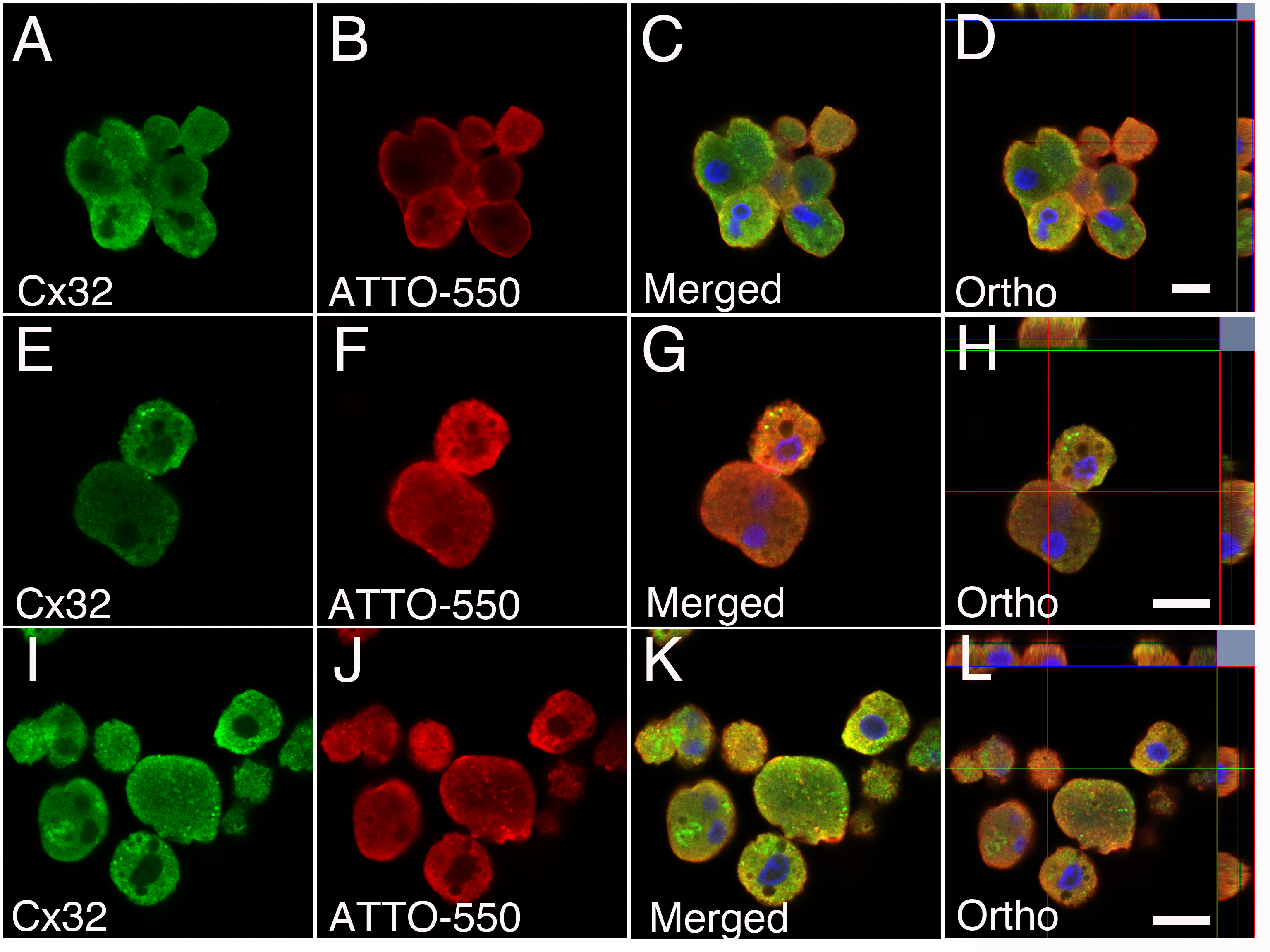

Supplement: Supplementary file 1 — Additional file 1: Figure 1. Primary human hepatocytes take up oligomeric α-syn assemblies in vitro. A–F) Confocal image analysis of primary hepatocytes incubated with ATTO-550 labeled oa-syn (red) and then immunolabeled with Cx32 (green) demonstrate the internalization of oα-syn and a partial co-localization between the gap junction protein Cx32 (yellow, orthogonal views). All cells were counterstained with DAPI (blue). Bars = 10 μm. [file 40478_2021_1136_MOESM1_ESM.tif]

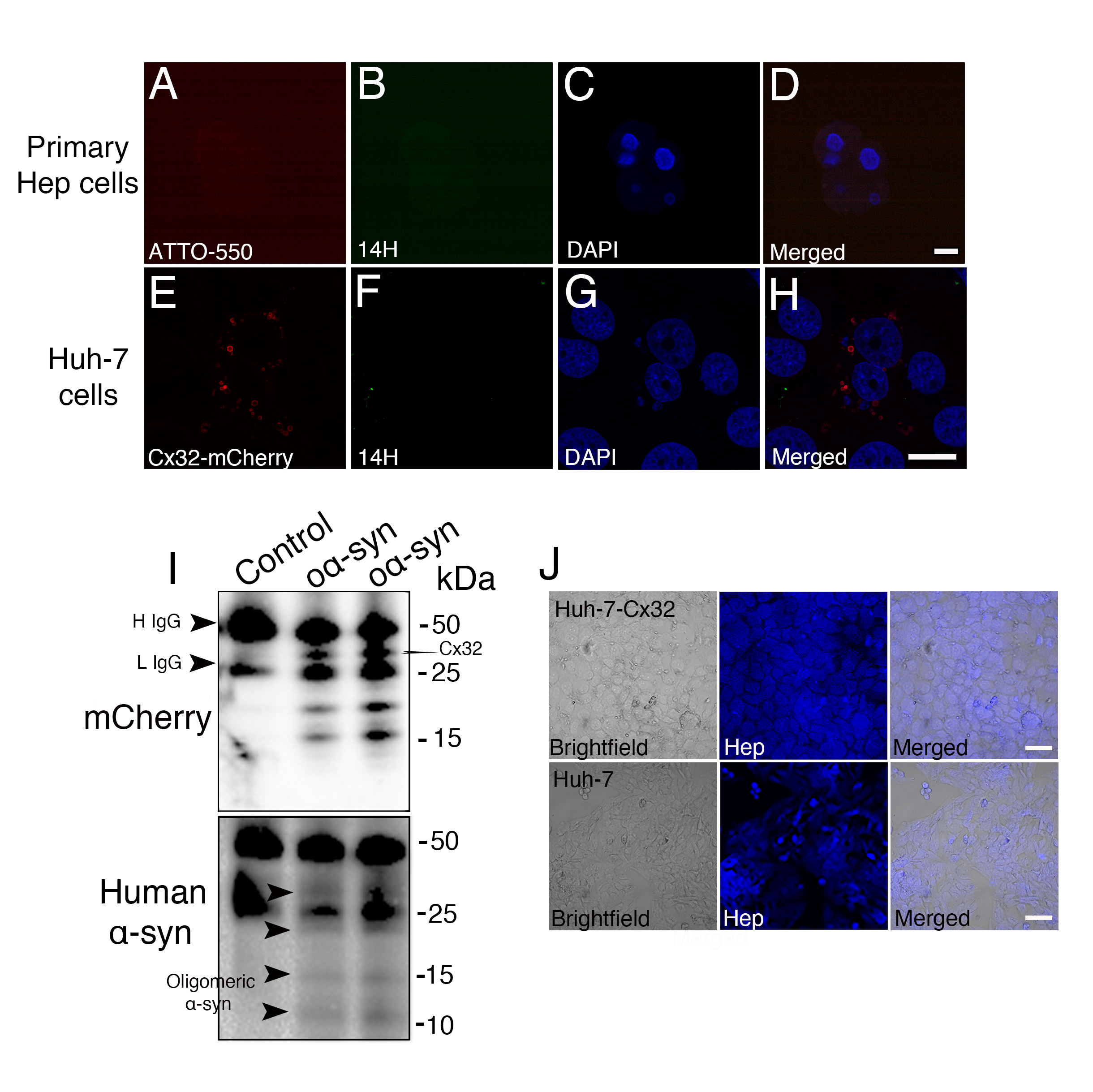

Supplement: Supplementary file 2 — Additional file 2: Figure 2. Immunoprecipitation of human α-syn oligomers pulls down Cx32. A–D) Immunolabeling on primary human hepatocytes or E–H) HuH-7 cells expressing Cx32 (red) using the human specific 14H antibody show no α-syn reactivity in the absence of α-syn treatment. I) Immunoprecipitation of human α-syn oligomers in HuH-7-Cx32 cells treated with α-syn oligomers pulls down Cx32 whereas untreated cells show no Cx32 pulldown and only the low and heavy chains of the antibody used are shown. J) Confocal image analysis of HuH-7 and HuH-Cx32 cells showing the morphology with or without Cx32 expression. Bars A–H = 20 μm and J= 50 μm. [file 40478_2021_1136_MOESM2_ESM.tif]

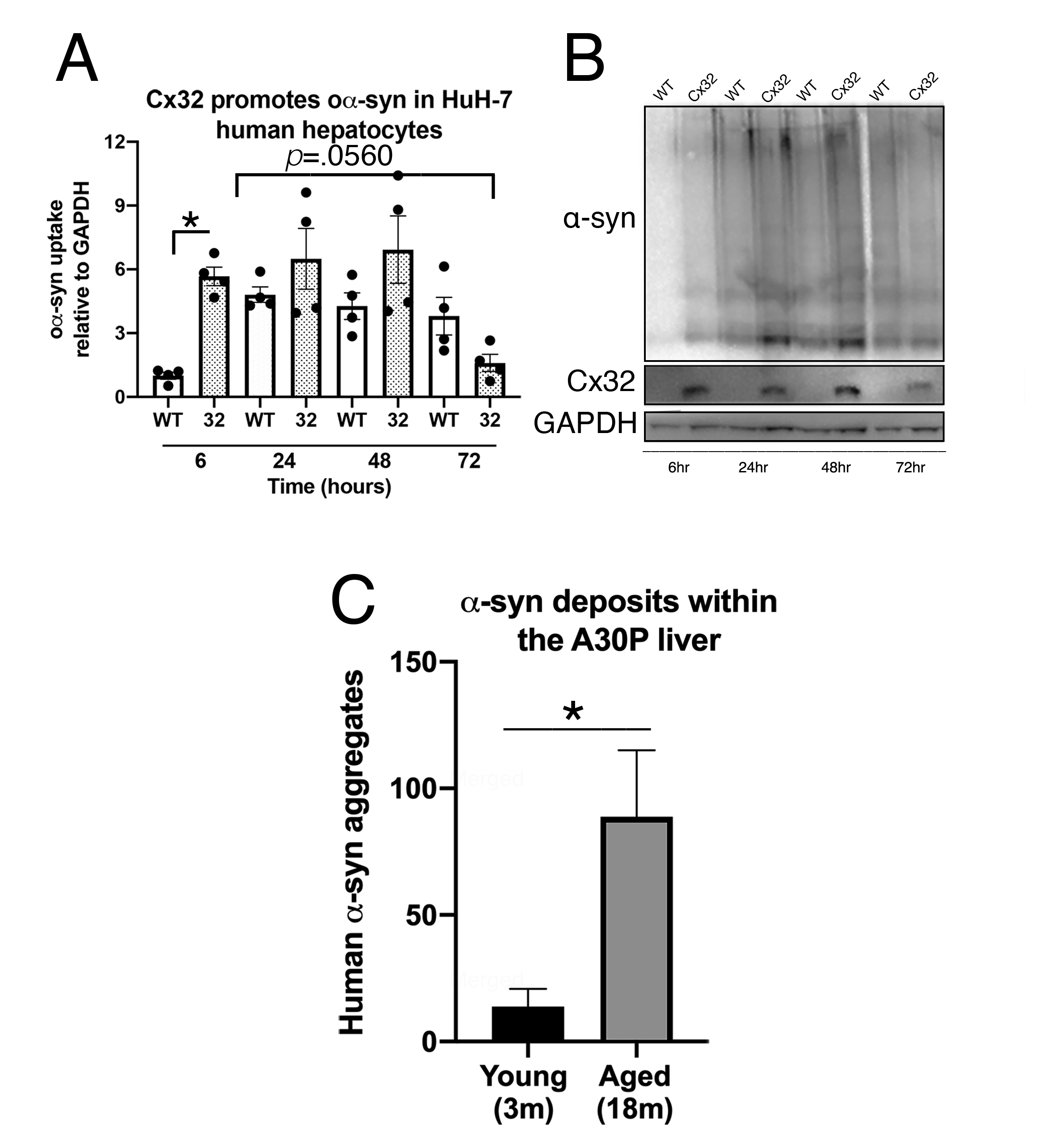

Supplement: Supplementary file 3 — Additional file 3: Figure 3. Cx32 expression in HuH-7 cells promote α-syn uptake. A, B) oα- syn uptake in WT HuH-7 cells (white bars) or HuH-7 cells expressing Cx32 (dotted bars) for different time periods using Western blot analysis. C) Quantification of human α-syn deposits in young (3 months) and aged (18 months) A30P liver tissue sections demonstrates a progressive accumulation α-syn over time. *p<0.05. [file 40478_2021_1136_MOESM3_ESM.tif]

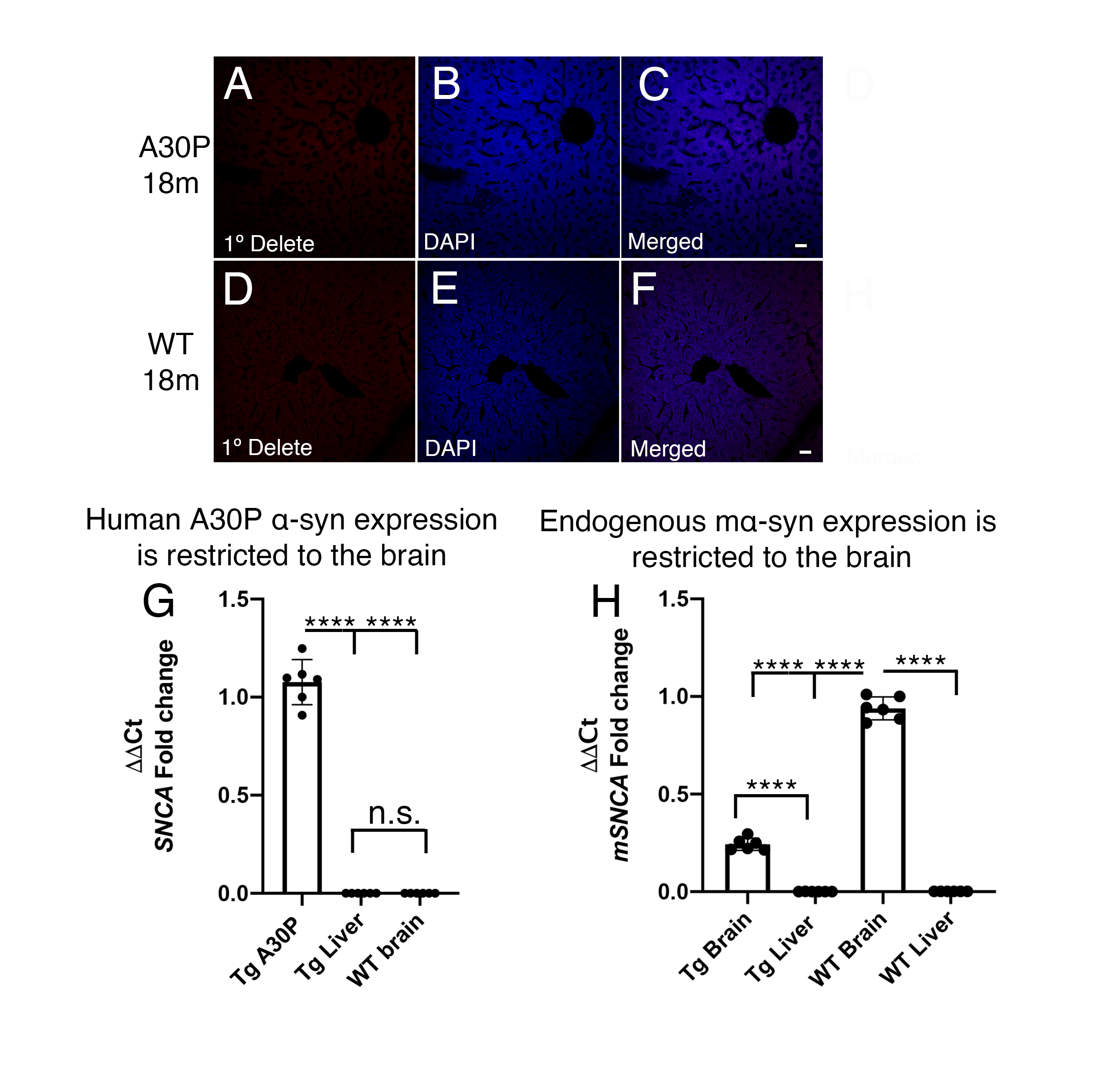

Supplement: Supplementary file 4 — Additional file 4: Figure 4. Human and mouse α-syn expression is restricted to the brain. A–C) Confocal image analysis of liver tissue sections from aged A30P show no immunoreactivity to α-syn D–F) WT livers in the presence or absence of primary antibody (14H) show no immunoreactivity to mouse α-syn. G) qRT-PCR analysis of human α-syn in A30P brain, liver and normal wild type brains. H) qRT-PCR analysis of endogenous mα-syn in A30P brain, liver and normal wild type brain and liver. n.s.= non-significant. ****p<0.0001, Bars = 20 μm. [file 40478_2021_1136_MOESM4_ESM.tif]

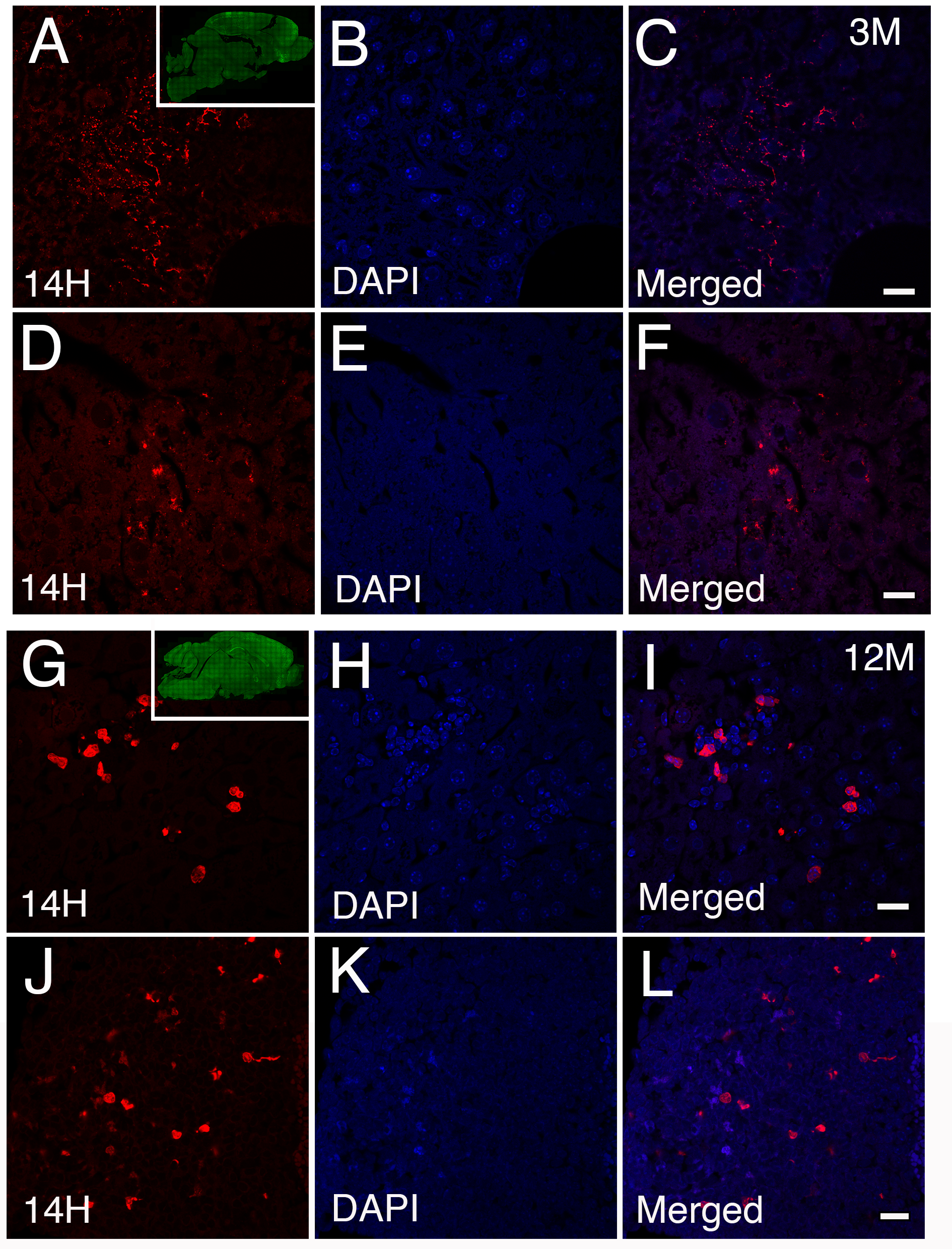

Supplement: Supplementary file 5 — Additional file 5: Figure 5. Age dependent accumulation of human α-syn deposits within the liver of the L61 model of PD. A–F) Confocal image analysis using the 14H antibody shows the presence of human α-syn deposits (red) in young liver sections (3 months) as small puncta located within the portal tracts and liver parenchyma. Insert within panel (A) shows the deposition of α-syn within the brain of the L61 mice at 3 months of age immunostained with pS129 antibodies (green). G–L) Aged liver tissue sections (12 months) showing the progressive accumulation of human α-syn deposits within the portal tracts and liver parenchyma within the L61 model. Insert within panel (H) shows the deposition of α-syn at 12 months of age immunostained with pS129 antibodies (green). All tissue sections were counterstained with DAPI (blue). Bars = 20 μm. [file 40478_2021_1136_MOESM5_ESM.tif]

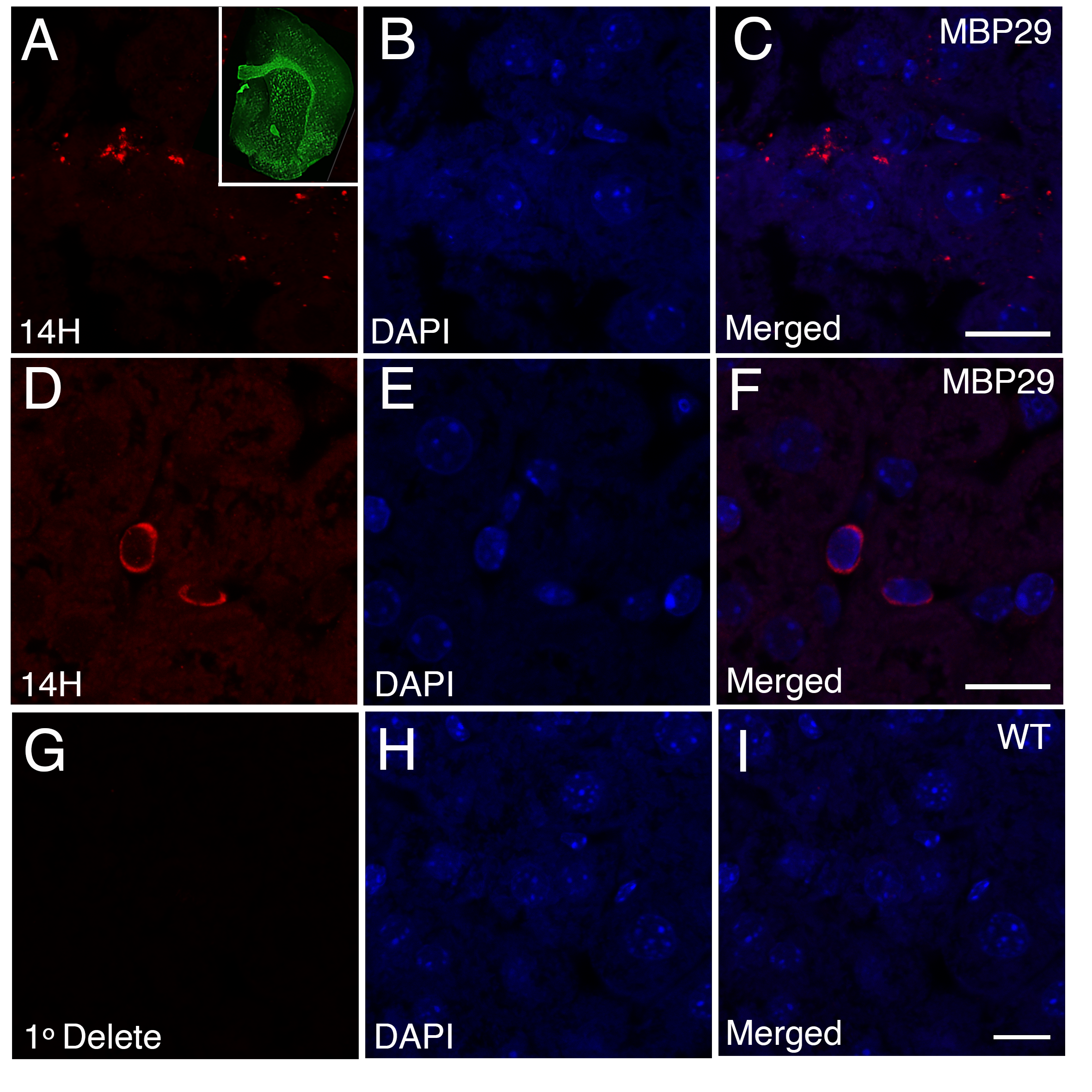

Supplement: Supplementary file 6 — Additional file 6: Figure 6. Identification of human α-syn deposits within the MBP29 liver modeling MSA. A–C) Confocal image analysis using the 14H antibody shows the presence of human α-syn deposits (red) in young liver sections (4 months) as small puncta located within the portal tracts and liver parenchyma. Insert within panel A shows the deposition of α-syn within the brain of the MBP29 mice at 4 months of age immunostained for total α-syn (green). D–F) In some instances, we identified the presence of human α-syn within the sinusoidal region likely surrounding inflammatory cells. G–I) Tissue sections lacking primary antibody (14H) show no human α-syn immunoreactivity. All tissue sections were counterstained with DAPI (blue). Bars = 20 μm. [file 40478_2021_1136_MOESM6_ESM.tif]

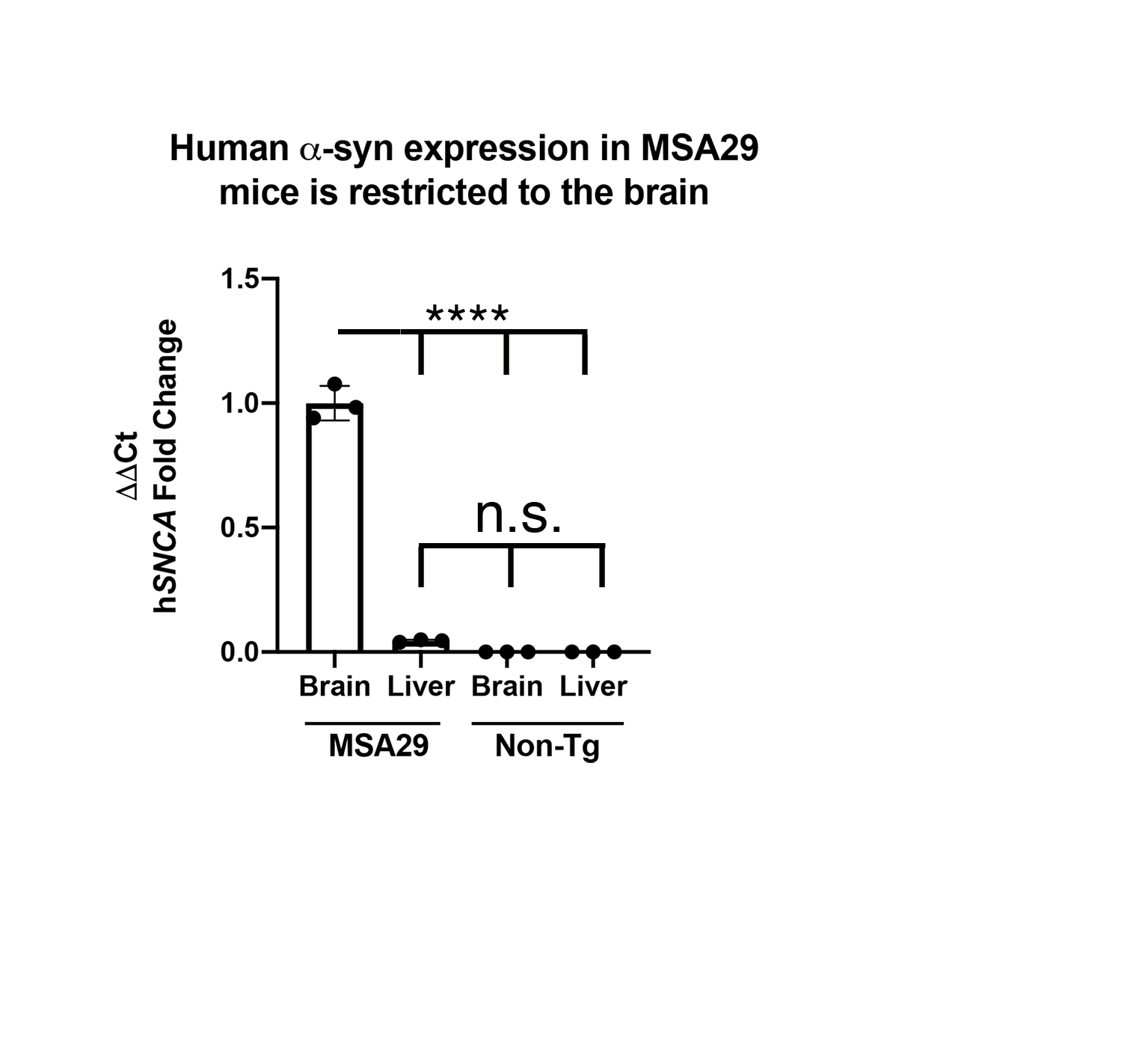

Supplement: Supplementary file 7 — Additional file 7: Figure 7. Human and mouse α-syn expression in MBP29 mice is restricted to the brain. A) qRT-PCR analysis of human α-syn in MBP29 brain, liver as well as non- transgenic (non-Tg) wild type brains and liver samples. n.s.= non-significant. ****p<0.0001. [file 40478_2021_1136_MOESM7_ESM.tif]

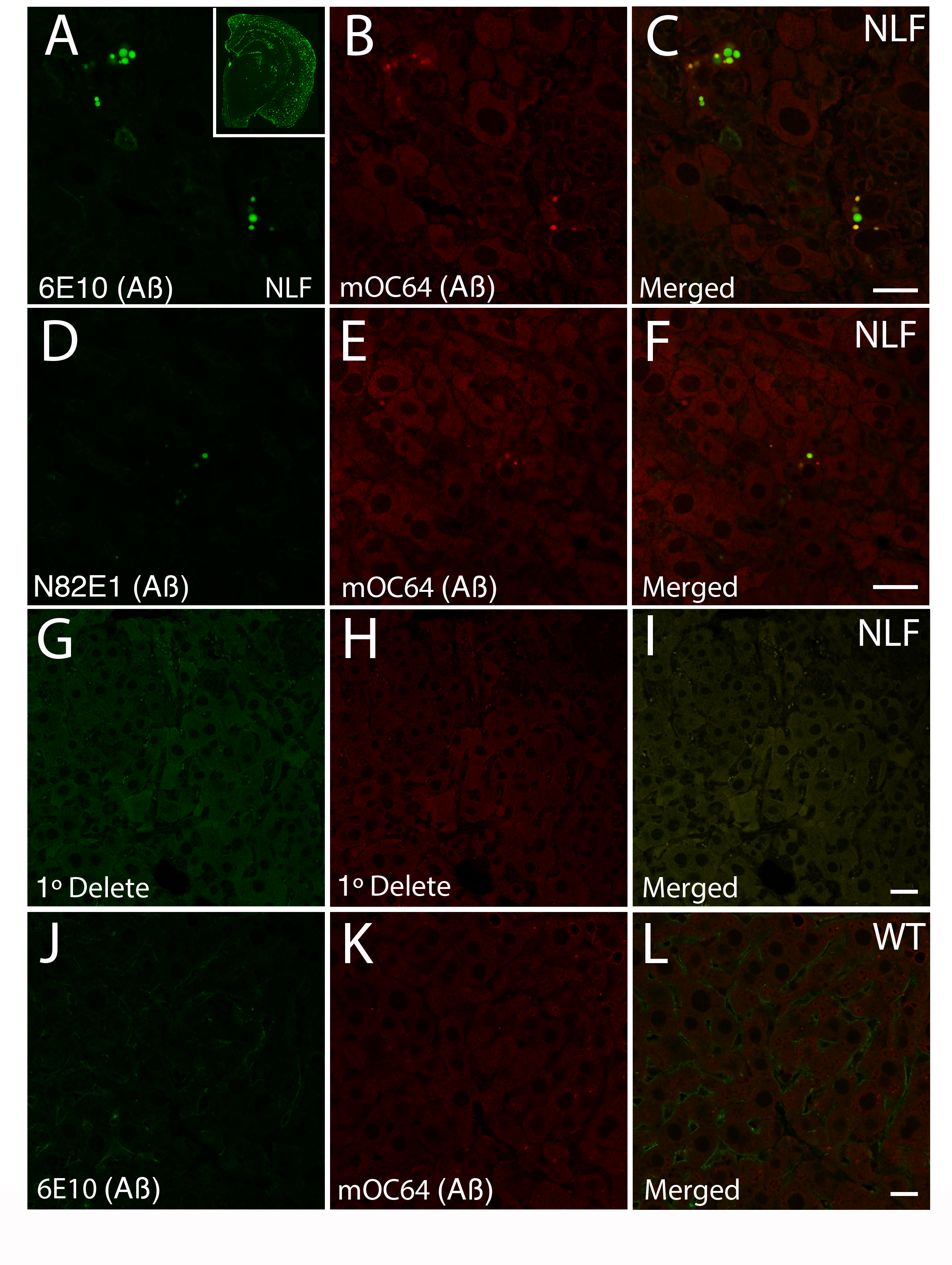

Supplement: Supplementary file 8 — Additional file 8: Figure 8. Identification of amyloid beta (Aß) deposits within the liver of NL-F mice liver modeling AD. A–C) Confocal image analysis of NL-F livers (24-month) using the antibody 6E10. Insert within panel A shows the deposition of α-syn within the brain of the NL-F mice at 24 months of age immune-stained with N82E antibodies (green). D–F) N82E shows the presence of human Aß inclusions (green) which co-localize with the rabbit monoclonal mOC65 antibody (red) within the liver parenchyma. G–I) Tissue sections lacking primary antibody (6E10, mOC64) show no Aß immunoreactivity. Bars = 20 μm. [file 40478_2021_1136_MOESM8_ESM.tif]

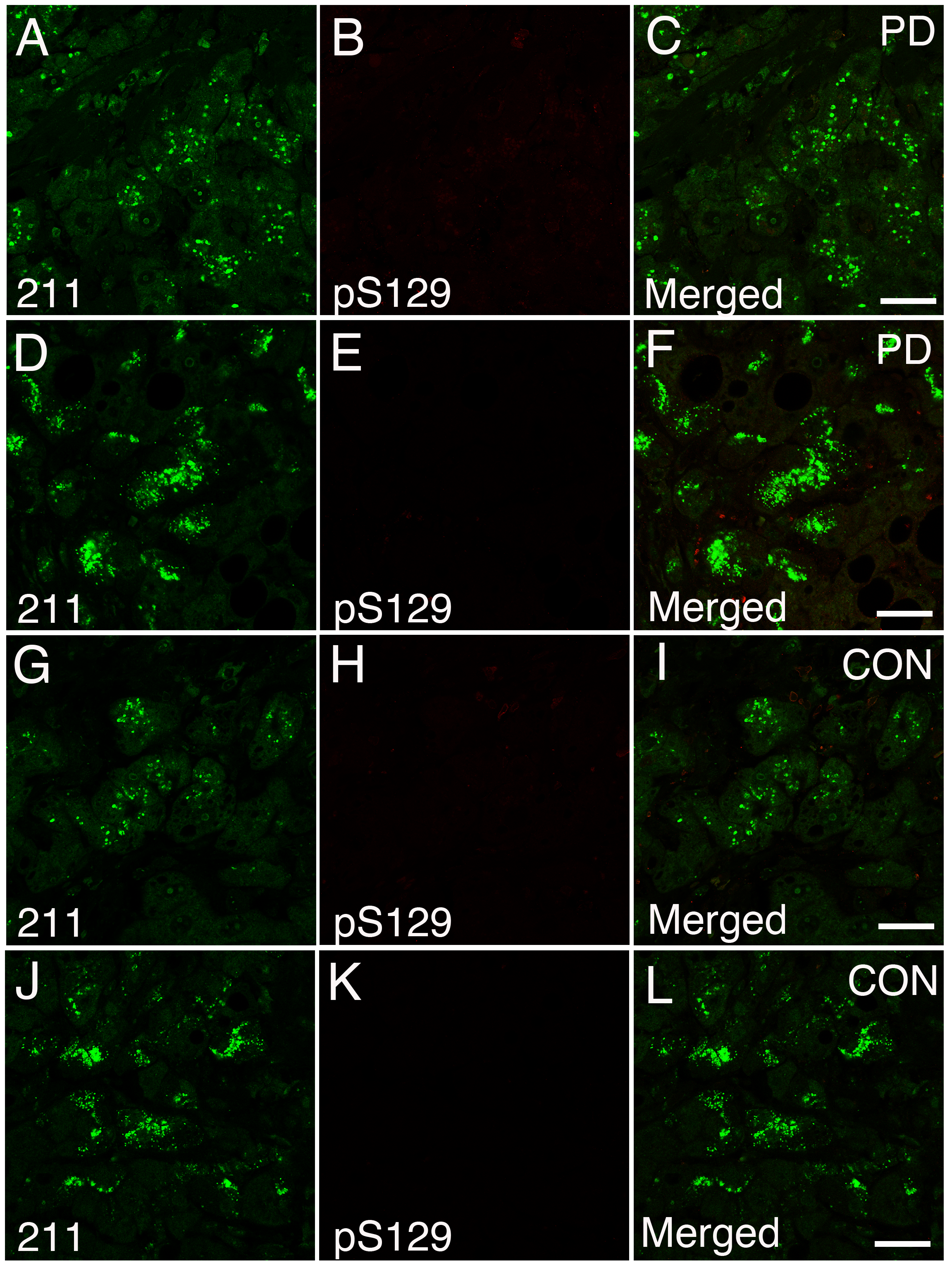

Supplement: Supplementary file 9 — Additional file 9: Figure 9. Accumulation of α-syn within the human liver lacks pS129 immunoreactivity. Confocal image analysis on human liver tissue sections immunolabeled with the 211 human-specific antibody (green) demonstrates the presence of human α-syn deposits within human hepatocytes, portal tracts, and liver parenchyma, however, negative for pS129 immunoreactivity both in PD (A–F) and aged-matched controls (CON) (G–L). Bars = 20 μm. [file 40478_2021_1136_MOESM9_ESM.tif]
