# Supplementary material for: Accumulation of alpha-synuclein within the liver, potential role in the clearance of brain pathology associated with Parkinson’s disease
Source: Acta Neuropathol Commun. 2021 Mar 20;9:46. doi: 10.1186/s40478-021-01136-3 (PMC7980682; doi:10.1186/s40478-021-01136-3)
Supplement: Supplementary file 10 — Additional file 10: Table I. Primary antibodies and primers used in this study. [file 40478_2021_1136_MOESM10_ESM.docx]

Supplemental Table I: Primary antibodies and primers used in this study

| Antibody | Clone | Company |
| --- | --- | --- |
| α-syn | 14H2L1 | Thermo Scientific |
| α-syn | 211 | Abcam |
| α-syn | D37A6 | Cell Signaling |
| α-syn | 15G7 | Enzo Life sciences |
| pS129 | psyn#64 | Wako Chemicals |
| pS129 | Ab59264 | Abcam |
| CD11 | Ab133357 | Abcam |
| CD45 | Ab10558 | Abcam |
| GFAP | Ab7260 | Abcam |
| F4/80 | 111101 | Abcam |
| Hepatocytes | OCH1E5 | Cell Marque |
| mCherry | PA5-34974 | Thermo Fisher |
| 6E10 (Aß) | 803004 | Biolegend |
| N82E1 (Aß) | JP10323 | IBL International |
| Aß | mOC64 | Abcam |

| ß-Tubulin | TUBB3 | Biolegend |
| --- | --- | --- |

| Genes | ID | Company |
| --- | --- | --- |
| hSNCA | Hs01103383 | Taqman™ |
| hSNCA | **F***GCAGGGAGCATTGCAGCAGC)  **R***GGCTTCAGGTTCGTAGTC)  **F***GGAGGGAGTTGTGGCTGCTGC  **R***GCCAGTGGCTGCTGCAATGC | Ebrahimi-Fakhari et al., 2011  Inhouse |
| mSNCA | Mm01188700 | Taqman™ |
| mGAPDH | Mm99999915 | Taqman™ |
| 18S | F*GGACCAGAGCGAAAGCATTTG  R*GCCAGTCGGCATCGTTTATG | Pernot et al., 2010 |

| Cx32 | MAB3069 | Sigma |
| --- | --- | --- |

F*= Forward Primer, R*= Reverse Primer
