# Supplementary material for: Accumulation of alpha-synuclein within the liver, potential role in the clearance of brain pathology associated with Parkinson’s disease
Source: Acta Neuropathol Commun. 2021 Mar 20;9:46. doi: 10.1186/s40478-021-01136-3 (PMC7980682; doi:10.1186/s40478-021-01136-3)
Supplement: Supplementary file 11 — Additional file 11: Table II. Description of animal models of neurodegeneration used in this study. [file 40478_2021_1136_MOESM11_ESM.docx]

**Supplemental Table II.** Description of animal models of neurodegeneration used in this study.

| Promoter | Line name | Protein | Human α-syn levels* | Average Survival (Months) | References |
| --- | --- | --- | --- | --- | --- |
| Thy-1 | A30P | hα-syn (A30P) | ~2X protein | ~18 | Kahle et al, 2000 |
| Thy-1 | L61 | hα-syn (WT) | ~10X mRNA | ~12-15 | Rockenstein et al., 2002 |
| MBP | MBP29 | hα-syn | ~3X protein | ~4.5 | Shults et al., 2005 |
| Endogenous | *App^NL-F^* | hAb  Beyreuther/  Iberian | Endogenous levels | Normal | Saito et al., 2014 |

*Relative to endogenous mouse α-syn levels
