# Supplementary material for: Accumulation of alpha-synuclein within the liver, potential role in the clearance of brain pathology associated with Parkinson’s disease
Source: Acta Neuropathol Commun. 2021 Mar 20;9:46. doi: 10.1186/s40478-021-01136-3 (PMC7980682; doi:10.1186/s40478-021-01136-3)
Supplement: Supplementary file 12 — Additional file 12: Table III. Liver characterization of animal models of PD and MSA. [file 40478_2021_1136_MOESM12_ESM.docx]

**Supplemental Table III.** Liver characterization of animal models of PD and MSA. Pathological characterization of livers modeling PD (L61, A30P), MSA (MBP29) or AD (*App^NL-F^*). The presence of liver pathology was determined using standard clinical criteria, graded 0-4.

| Strain | n number | Sex | Age, Months | Liver  α-syn depositon | Fibrosis | Steatosis | Liver  inflammation |
| --- | --- | --- | --- | --- | --- | --- | --- |
| WT | 7 | M | 3 | 0 | 0 | 0 | 0 |
| WT | 7 | M | 18 | 0 | 0 | 0 | 0 |
| A30P | 8 | M | 3 | 2 | 0 | 0 | 1 |
| A30P | 12 | M | 18 | 4 | 0 | 0 | 4 |
| WT | 4 | M | 3 | 0 | 0 | 0 | 0 |
| WT | 4 | M | 12 | 0 | 0 | 0 | 0 |
| L61 | 6 | M | 3 | 1 | 0 | 0 | 0 |
| L61 | 8 | M | 12 | 2 | 0 | 0 | 0 |
| WT | 4 | M | 4 | 0 | 0 | 0 | 0 |
| MBP29 | 4 | M | 4 | 1 | 0 | 0 | 0 |
| WT | 4 | M | 20 | 0 | 0 | 0 | 0 |
| *App^NL-F^* | 4 | M | 20 | 0 | 0 | 0 | 0 |
| WT | 2 | M | 24 | 0 | 0 | 0 | 0 |
| *App^NL-F^* | 2 | M | 24 | 0 | 0 | 0 | 0 |
