# Supplementary material for: Accumulation of alpha-synuclein within the liver, potential role in the clearance of brain pathology associated with Parkinson’s disease
Source: Acta Neuropathol Commun. 2021 Mar 20;9:46. doi: 10.1186/s40478-021-01136-3 (PMC7980682; doi:10.1186/s40478-021-01136-3)
Supplement: Supplementary file 13 — Additional file 13: Table IV. Clinical and pathological characterization of human cases included in this study. [file 40478_2021_1136_MOESM13_ESM.docx]

**Supplemental Table IV.** Clinical and pathological characterization of human cases included in this study. Brain α-syn pathology was determined at autopsy after the specified post mortal delay (PMD) as Lewy body disease (LBD), mixed Alzheimer’s and Parkinson’s disease (AD/PD), Dementia with Lewy bodies (DLB), Unspecified dementia (DEM), Cerebrovascular disease (CeVD) or free from brain pathology (none). Brain pathology was classified as ADNC (Alzheimer’s Disease Neuropathologic Change) and/or PART (Primary Age related tauopathy). The presence of liver pathology was determined using standard clinical criteria, graded 0-4.

| Case | Clinical Diag | Brain  path  LBD | Brain path  PART/ADNC | Final brain Path | Sex | Age | PMD  (Brain and Liver) | Liver α-syn path | Fibrosis | Steatosis | Liver Inflammation | Cholestasis |
| --- | --- | --- | --- | --- | --- | --- | --- | --- | --- | --- | --- | --- |
| 1 | PD | stage 5 | PART | LBD | M | 67 | 96 | Yes | 0 | 2 | 0 | 0 |
| 2 | PD | stage 5 | PART | LBD | F | 80 | 48 | No | 0 | 3 | 1 | Yes |
| 3 | PD | stage 6 | PART | LBD | F | 78 | 48 | Yes | 2 | 0 | 2 | Yes, light |
| 4 | PD | stage 5 | PART | LBD | M | 77 | 96 | Yes | 0 | 0 | 0 | Yes, light |
| 5 | PD | stage 5 | ADNC Intermedita | MIXED | M | 79 | 96 | Min, Hep | 0 | 0 | 0 | Yes |
| 6 | PD | stage 6 | PART | LBD | M | 82 | 96 | No | 0 | 0 | 0 | Yes |
| 7 | PD | stage 5 | ADNC Intermedita | MIXED | F | 79 | 48 | Min, Hep | 0 | 0 | 0 | Yes |
| 8 | PD | stage 6 | ADNC low | LBD | M | 76 | 24 | Yes, Hep | 0 | 1 | 0 | Yes, focally |
| 9 | AD/PD | stage 6 | ADNC low | LBD | F | 69 | 72 | No | 0 | 0 | 0 | Yes |
| 10 | DLB | stage 6 | ADNC intermediate | MIXED | M | 78 | 24 | Yes, Hep | 0 | 0 | 0 | Yes |
| 11 | DEM | stage 6 | PART | LBD | F | 93 | 96 | Yes | 0 | 2 | 0 | Yes |
| 12 | DEM | stage 6 | ADNC intermediate | MIXED | M | 75 | 72 | No | 0 | 0 | 0 | Yes |
| 13 | DEM | stage 5 | PART | LBD | M | 72 | 92 | Min, Hep | 0 | 0 | 0 | Yes, focally |
| 14 | CeVD | stage 5 | PART | LBD | F | 86 | 24 | Yes | 1 | 0 | 1-2 | Yes |
| 15 | CeVD | stage 5 | PART | LBD | F | 82 | 96 | Yes | 0 | 1 | 0 | Yes, light |
| 16 | CeVD | stage 5 | ADNC intermediate | AD | M | 82 | 48 | Yes, Hep | 0 | 0 | 0 | 0 |
| 17 | CeVD | none | ADNC low | CeVD | F | 80 | 72 | Yes, Hep | 0 | 0 | 0 | Yes |
| 18 | CeVD | none | PART | CeVD | M | 79 | 48 | Yes, Hep | 0 | 1 | 0 | Yes |
| 19 | CeVD | none | ADNC low | CeVD | F | 86 | 24 | Yes | 2-3 | 3 | 0-1 | 0 |
| 20 | CeVD | none | ADNC intermediate | AD | M | 82 | 72 | Yes | 3 | 0 | 0 | Yes |
| 21 | CeVD | none | ADNC low | CeVD | F | 82 | 96 | Min, Hep | 0 | 0 | 0 | Yes |
| 22 | CeVD | none | PART | CeVD | M | 75 | 48 | No | 0 | 0 | 0 | Yes |
| 23 | CeVD | none | none | CeVD | M | 78 | 72 | No | 0 | 2 | 0 | 0 |
| 24 | None | none | none | CONT | M | 82 | 48 | Yes | 0 | 1 | 0 | Focally |
| 25 | None | none | none | CONT | M | 67 | 72 | Yes, Hep | 1 | 2 | 0 | 0 |
| 26 | None | none | none | CONT | F | 93 | 24 | Yes | 0 | 0 | 0 | Yes |
| 27 | None | none | PART | PART | F | 69 | 24 | No | 0 | 0 | 0 | Yes |
| 28 | None | none | ADNC intermediate | ADpreclin | M | 78 | 96 | No | 2 | 3 | 0 | 0 |
| 29 | None | none | PART | PART | F | 79 | 48 | No | 0 | 2 | 0 | Yes |
| 30 | None | none | PART | PART | M | 77 | 24 | No | 0 | 2-3 | 0 | 0 |

Hep: Hepatocytes, Min: Minimal
